# CD24^+^LCN2^+^ liver progenitor cells in ductular reaction contributed to macrophage inflammatory responses in chronic liver injury

**DOI:** 10.1186/s13578-023-01123-2

**Published:** 2023-10-02

**Authors:** Wei-Jian Huang, Bi-Jun Qiu, Xiao-Shu Qi, Cai-Yang Chen, Wen-Ming Liu, Shen-ao Zhou, Min Ding, Feng-Feng Lu, Jie Zhao, Dan Tang, Xu Zhou, Gong-Bo Fu, Zhen-Yu Wang, Hong-Qian Ma, Yu-Ling Wu, Hong-ping Wu, Xiao-Song Chen, Wei-Feng Yu, He-Xin Yan

**Affiliations:** 1 Department of Anesthesiology and Critical Care Medicine, School of Medicine, Renji Hospital, Shanghai Jiaotong University, Shanghai, 200120 China; 2 Key Laboratory of Anesthesiology (Shanghai Jiao Tong University), Ministry of Education, Shanghai, China; 3 Celliver Biotechnology Inc., Shanghai, China; 4 Department of Liver Surgery, Renji Hospital, School of Medicine, Shanghai Jiao Tong University., Shanghai, China; 5 Institute for Regenerative Medicine, Shanghai East Hospital, School of Life Sciences and Technology, Tongji University, Shanghai, China; 6 Department of Interventional Oncology, Renji Hospital, School of Medicine, Jiaotong University, Shanghai, China; 7 Department of Medical Oncology, First School of Clinical Medicine, Jinling Hospital, Southern Medical University, Nanjing, China; 8 State Key Laboratory of Oncogenes and Related Genes, School of Medicine, Renji Hospital, Shanghai Cancer Institute, Shanghai Jiaotong University, Shanghai, China; 9 International Cooperation Laboratory On Signal Transduction, Eastern Hepatobiliary Surgery Hospital, Second Military Medical University, Shanghai, China; 10 Department of Infectious Diseases, Renji Hospital, Shanghai Jiaotong University School of Medicine, Shanghai, 200120 China

**Keywords:** Resident liver progenitor cells, Small molecule cocktail, Ductular reaction, Macrophages, LCN2

## Abstract

**Background:**

CD24^+^CK19^+^/CD24^+^SOX9^+^ resident liver cells are activated and expanded after chronic liver injury in a ductular reaction. However, the sources and functions of these cells in liver damage remain disputed.

**Results:**

The current study combined genetic lineage tracing with in vitro small-molecule-based reprogramming to define liver progenitor cells (LPCs) derived from hepatic parenchymal and non-parenchymal tissues. tdTom^+^ hepatocytes were isolated from *ROSA26*^*tdTomato*^ mice following AAV8*-Tbg-Cre*-mediated recombination, EpCAM^+^ biliary epithelial cells (BECs) from wild-type intrahepatic bile ducts and ALB/GFP^−^EpCAM^−^ cells were isolated from *Alb*^*CreERT*^*/R26*^*GFP*^ mice. A cocktail of small molecules was used to convert the isolated cells into LPCs*.* These in vitro cultured LPCs with CD24 and SOX9 expression regained the ability to proliferate. Transcriptional profiling showed that the *in-vitro* cultured LPCs derived from the resident LPCs in non-parenchymal tissues expressed Lipocalin-2 (*Lcn2*) at high levels. Accordingly, endogenous *Cd24a*^+^*Lcn2*^+^ LPCs were identified by integration of sc-RNA-sequencing and pathological datasets of liver dysfunction which indicates that LPCs produced by ductular reactions might also originate from the resident LPCs. Transplantation of *in-vitro* cultured *Cd24a*^+^*Lcn2*^+^ LPCs into CCl_4_-induced fibrotic livers exacerbated liver damage and dysfunction, possibly due to LCN2-dependent macrophage inflammatory response.

**Conclusions:**

CD24^+^LCN2^+^ LPCs constituted the expanding ductular reaction and contributed to macrophage-mediated inflammation in chronic liver damage. The current findings highlight the roles of LPCs from distinct origins and expose the possibility of targeting LPCs in the treatment of chronic hepatic diseases.

**Graphical Abstract:**

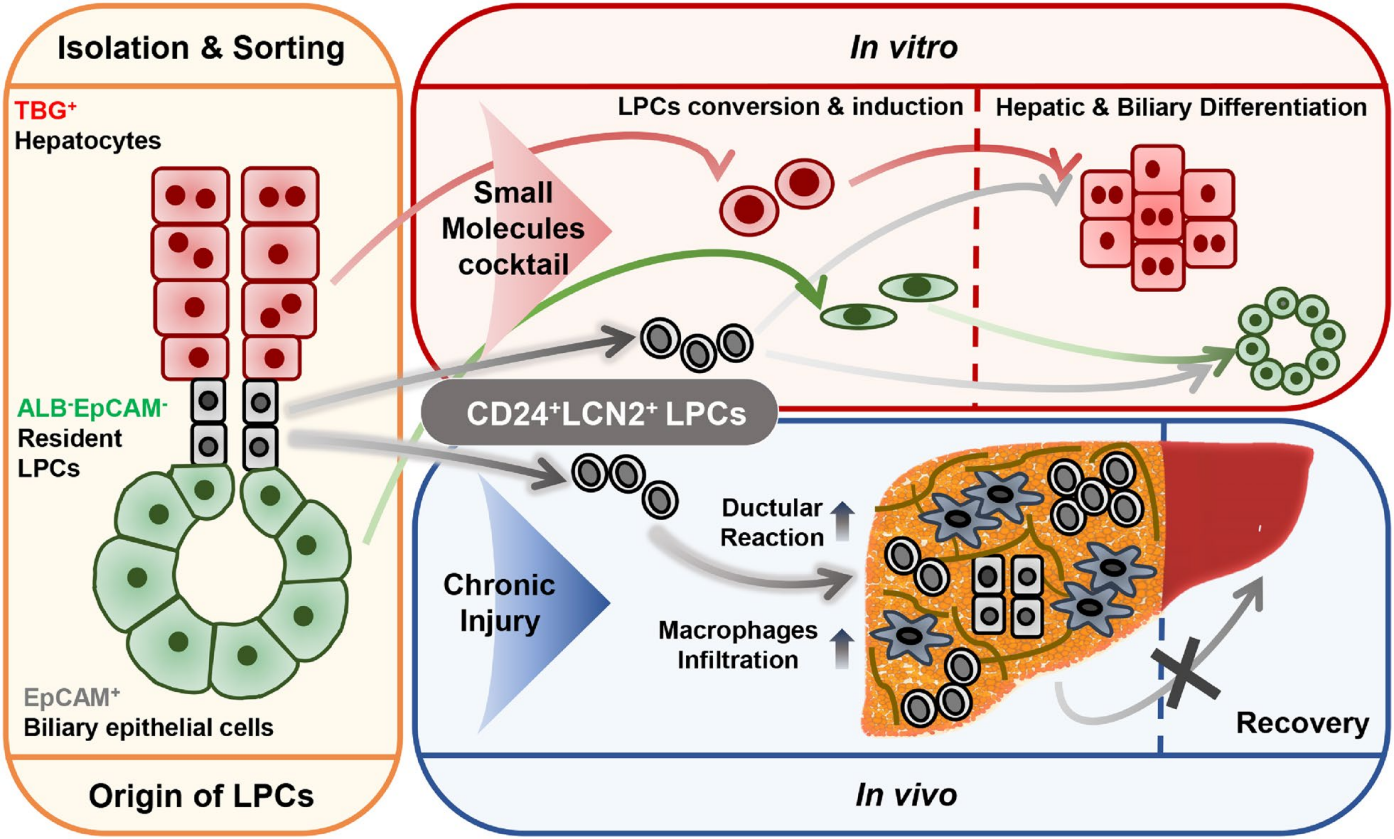

**Supplementary Information:**

The online version contains supplementary material available at 10.1186/s13578-023-01123-2.

## Introduction

The liver possesses immense regenerative potential. Chronic or severe liver damage including liver fibrosis and non-alcoholic steatohepatitis (NASH), is accompanied by pathological proliferation or hyperplasia of the bile duct, referred to as the ductular reaction [1]. These ductular reactive cells express intermediate hepato-biliary markers and expand from the periportal region into the surrounding parenchyma, which represents the activation of facultative liver progenitor cells (LPCs) [1, 2]. Recently, much attention has been paid to the precise origin and role of these hepatic injury-induced LPCs in ductular reaction [3, 4], especially concerning advanced liver diseases.

Cells located in the canals of Hering of the adult liver are considered to be the resident LPCs and express markers of both fetal hepatocytes and biliary epithelial cells (BECs), which may generate hepatocytes and bile duct cells during chronic injury [5, 6]. Recent studies have demonstrated the potential of both hepatocytes and BECs for post-injury division [7, 8] and mutual transdifferentiation [9]. Thioacetamide-induced liver damage [10] or genetically impaired hepatocyte proliferation [11] have been shown to stimulate the conversion of BECs into bi-phenotypic states, capable of transdifferentiation into hepatocytes. In addition, the conversion of hepatocytes into LPCs was shown to promote liver parenchyma regeneration after chronic injury in hepatocyte fate tracing [12, 13] and chimeras of the fumarylacetoacetate hydrolase knockout (*Fah*^*−/−*^) mouse [7, 14]. Further studies indicated that LPCs originating from hepatocytes may be a predominant source of new parenchymal cells in mice undergoing chronic liver injury [15, 16]. These findings raised the question of which type of LPC contributes to the ductular reaction and how it affects liver repair.

We have previously assembled a small-molecule cocktail by mimicking the in vivo milieu of liver injury and regeneration that converts hepatocytes into expandable LPCs [17, 18]. Here, the proliferative activity of the resident LPCs was activated by the cocktail (CD24^+^LCN2^+^ LPCs), and their phenotypes and roles in liver dysfunction was characterized together with those of hepatocyte-derived LPCs (HepLPCs) and BEC-derived LPCs (BecLPCs). Transcriptional profiling and immunofluorescence staining allowed the identification of *Cd24a*^+^*Lcn2*^+^ LPCs as the major LPC type contributing to ductular reaction. Transplantation of *in-vitro* cultured *Cd24a*^+^*Lcn2*^+^ LPCs was found to exacerbate liver dysfunction by provoking a robust macrophage response via LCN2. These findings shed light on hepatic cellular plasticity and contribute to advances in LPC-based treatment for chronic liver disease.

## Results

### Generation and characterization of CD24^+^ liver progenitor cells from hepatic parenchymal and non-parenchymal cells

**Fig. 1:**
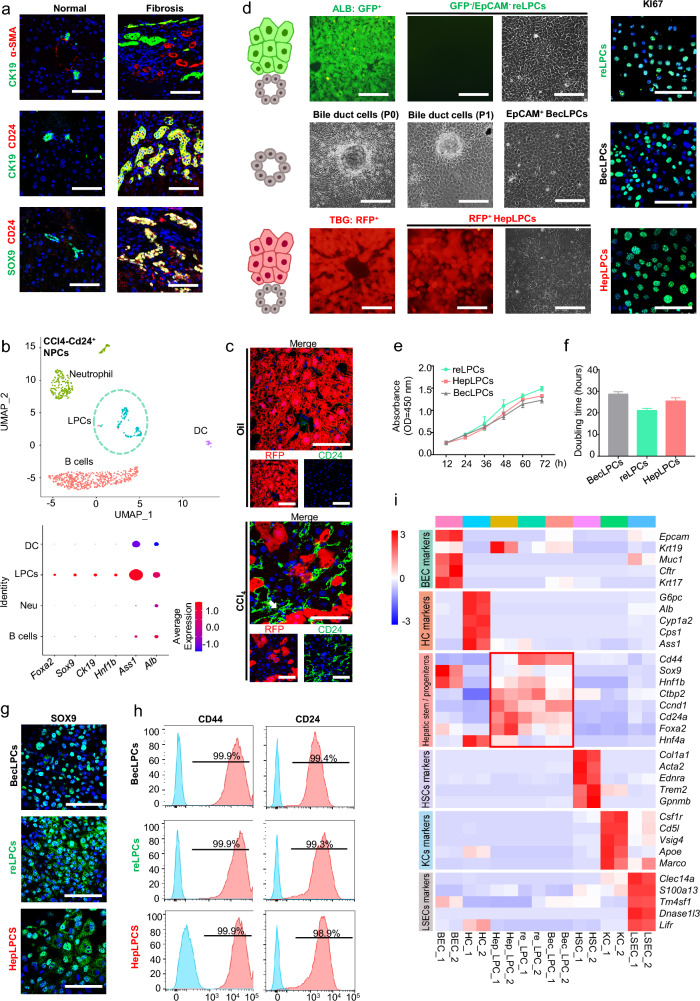
Derivation of cultured liver progenitor cells from the hepatic parenchymal and non-parenchymal cells. **a** Representative immunofluorescence staining of α-SMA, CK19, SOX9, and CD24 in human liver fibrotic tissues. Scale bar: 200 μm. **b** Liver non-parenchymal cells (NPCs) were isolated from mouse livers after 4 CCl_4_ injections and subjected to scRNA-Seq. U-map visualization of CD24^+^ cell clusters is based on 1038 single-cell transcriptomes and bubble plots show the expression levels of *Foxa2*, *Sox9*, *Ck19*, *Hnf1b*, *ASS1*, and *Alb*. The color bar indicates the expression level of scaled genes. **c** Representative immunofluorescence staining of CD24^+^tdTom^−^ liver progenitor cells in mice treated with CCl_4_. ROSA26^tdTomato^ mice were infected with AAV8-Tbg-Cre and treated with 2 mL/kg CCl_4_ twice a week for a continuous period of 6 weeks. Scale bar: 100 μm. **d** Schema model and representative images for isolation and culture of LPCs. ALB/GFP^−^EpCAM^−^ cells were isolated from Alb^CreERT^/R26^GFP^ mice by FACS and cultured in TEM (resident LPCs-derived LPCs). Mechanically abraded epithelial cells from wild-type intrahepatic bile ducts were FACS-sorted and EpCAM^+^ cells were cultured in TEM (BECs-derived LPCs). Control tdTom^+^ hepatocytes from ROSA26^tdTomato^ mice with AAV8-Tbg-Cre were cultured in TEM (Hepatocytes-derived LPCs). Scale bar: 200 μm. Representative immunofluorescence staining of Ki67 (Green) in three cultured LPCs at passage 5. Scale bar: 100 μm. **e** Proliferation measurements of progenitor-like cells in TEM calculated from CCK-8 assay at passage 5. **f** Progenitor-like cell doubling time at passage 5, measured by cell counting. **g** Representative immunofluorescence staining of SOX9 (Green) in three cultured LPCs at passage 5. Scale bar: 100 μm. **h** Quantification of CD44 and CD24 positive cells among three cultured LPCs at passage 5, assessed by flow cytometry. Red peaks represent staining samples and blue peaks represent the isotype control. **i** Heat map of three cultured LPCs of gene expression levels compared with HCs, BECs, HSCs, LSECs, and KCs. n = 2 independent experiments. Resident LPCs-derived LPCs, reLPCs; Hepatocytes-derived LPCs, HepLPCs; BECs-derived LPCs, BecLPCs; HC, hepatocyte; BEC, biliary epithelial cell; LSEC, liver sinusoidal endothelial cell; HSC, hepatic stellate cell; KCs, Kupffer cells. For panels e and f, data summarize 3 independent experiments. Data are expressed as means ± SD

CD24 is an essential marker of epithelial stem/progenitor cells, regulating homeostatic cell renewal by controlling the balance between proliferation and differentiation [19, 20]. In clinical samples of liver fibrosis, CD24^+^CK19^+^/CD24^+^SOX9^+^ ductular reactive cells were abundantly found in regions of ductular reaction (Fig. 1a, Additional file 1: Fig. S1a, Tables S4 and S5) [21], suggesting the activation and expansion of ductular reactive cells[1]. Consistently, sc-RNA analysis of NPCs after CCl_4_-induced liver injury identified a subset of facultative CD24^+^LPCs cells expressing SOX9/CK19/HNF1B/FOXA2/ALB/ASS1 (Fig. 1b) [22]. These findings confirmed the presence of the putative LPCs in ductular reaction. To determine the origin of LPCs in the damaged liver, *AAV-Tbg-Cre* infected *ROSA26*^*tdTomato*^ mice were used, in which hepatocytes and their derivatives were tdTomato-positive (tdTom^+^). With ductular reaction appearing after 6 weeks of exposure to CCl_4_ [23], an increasing population of CD24^+^tdTom^−^ LPCs was observed in ductular reactions foci (Fig. 1c, white arrow), suggesting that they originated from non-hepatocytes.

To further address their origin, a small molecule-based culture system, transition, and expansion medium (TEM) to culture hepatocyte-derived LPCs (HepLPCs) [17, 18], was used to culture LPCs derived from non-hepatocytes such as BECs or resident LPCs. Firstly, tdTom^+^ HepLPCs from *AAV-Tbg-Cre* infected *ROSA26*^*tdTomato*^ mice were included as controls (Fig. 1d, lower row). Next, to obtain biliary epithelial cells, intrahepatic bile ducts were digested and EpCAM^+^ BEC were isolated and expanded with a dedifferentiation process in TEM, giving rise to BEC-derived LPCs (BecLPCs) (Fig. 1d, middle row). Due to the lack of specific markers for LPCs in the canal of Hering [5], the *Alb*^*CreERT*^*/R26*^*GFP*^ strain was employed to yield GFP^−^ NPCs as almost all parenchymal cells of hepatic lineage were labeled with GFP. The labeling efficiency of Alb^CreERT^/R26^GFP^ mice by FACS was over 99% (Additional file 1: Fig. S1b). EpCAM^−^ cells were further isolated from GFP^−^cells to exclude the limited contamination by cholangiocytes. Then the purified ovoid cells were cultured and expanded in TEM (Fig. 1d, upper row). We assumed that these TEM-cultured proliferative cells originated from the pre-existing resident LPCs as previously reported [24].

All 3 types of LPCs grew to form continuous monolayers with similar doubling times at passage 5 (Fig. 1e, f). EdU incorporation assay and Ki67 staining indicated that all LPCs had similar division rates when cultured in TEM (Fig. 1d, Additional file 1: Figs. S1c, d). Immunofluorescence staining and FACS analysis showed that all three LPCs had similar expression levels of SOX9, CD24, and CD44, but not CD34, CD45, and CD90 (Fig. 1g, h, Additional file 1: Fig. S1d–f). Global gene expression analysis was conducted to compare the gene expression patterns of LPCs with those of Kupffer cells (KCs), hepatic stellate cells (HSCs), liver sinusoidal endothelial cells (LSECs), hepatocytes (HCs), and biliary epithelial cells (BECs) to exclude possible contamination by these cells (Fig. 1i). It is noteworthy that all three types of LPC expressed many stem/progenitor markers, such as *CD44*, *Hnf1b*, *Foxa2,* and *Sox9* [25]*.* In summary, these three cultured LPCs from different liver origins were generated by culture in TEM.

### Functional difference between LPCs from parenchymal cells and those derived from pre-existing LPCs

**Fig. 2:**
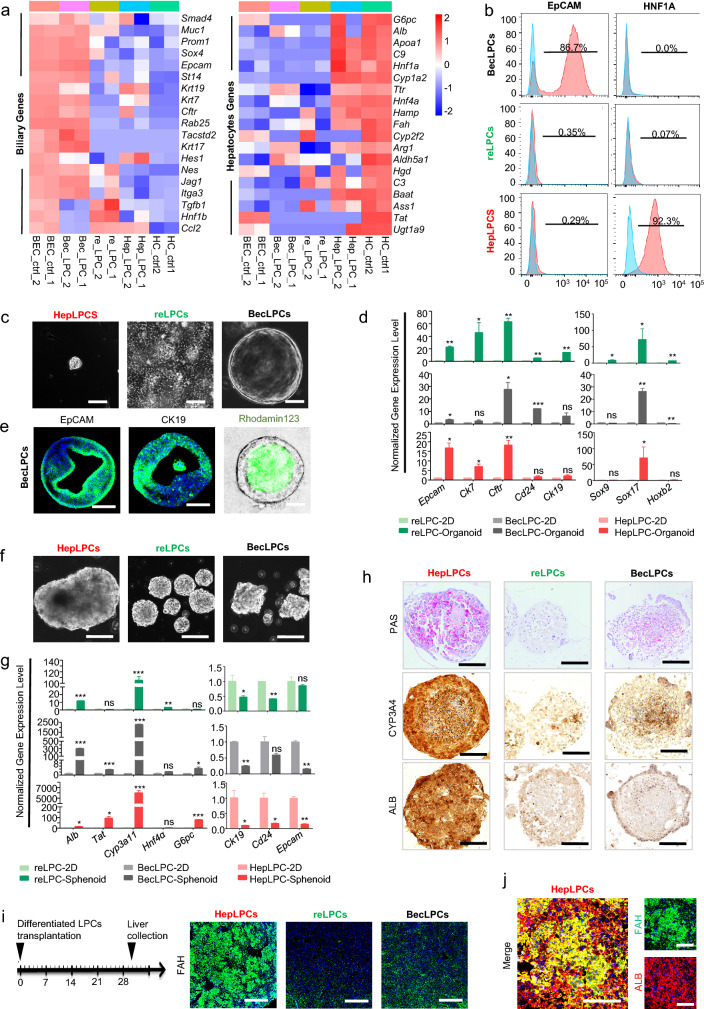
Three cultured liver progenitor cells are functionally distinct in vitro & in vivo. **a** Heat map showing known hepatocyte and biliary gene expression in three cultured LPCs, HC, and BEC. **b** Quantification of HNF1A and EpCAM positive cells among three cultured LPCs, assessed by flow cytometry. Red peaks represent staining samples and blue peaks represent the isotype control. **c** Representative images of organoids formed by three cultured LPCs. Scale bars, 100 μm. **d** Normalized expression levels of bile duct markers genes in LPCs derived organoids, analyzed by RT-q-PCR. **e** Representative immunofluorescence staining of EpCAM and CK19 and uptake of Rhodamine 123 dye in organoids formed by BecLPCs at day 10. Scale bars, 100 μm. **f** Representative images of spheroids formed by three cultured LPCs. Scale bars, 100 μm. **g** Normalized expression levels of hepatic and progenitor marker genes in LPCs derived organoids, analyzed by RT-q-PCR. **h** Representative PAS staining, and immunohistochemical staining for CYP3a4 and ALB of 3D spheroid formed by three cultured LPCs, respectively. Scale bars, 100 μm. **i** Schematic overview of the experimental design. The repopulated three cultured LPCs were analyzed by immunofluorescence staining for FAH expression, after transplantation of LPCs into Fah^−/−^ mice at day 30, as described in the Method. Scale bars, 600 μm. **j** The maturation of repopulated HepLPCs was analyzed by co-immunofluorescence staining for FAH and ALB expression at day 30 after transplantation of LPCs into Fah-/- mice, as described in the Method. Scale bars, 100 μm. Resident LPCs-derived LPCs, reLPCs; Hepatocytes-derived LPCs, HepLPCs; BECs-derived LPCs, BecLPCs. For panels **d** and **g**, results are shown as the mean ± S.D. of three independent experiments. *P < 0.05; **P < 0.005; ***P < 0.001.

RNA-seq demonstrated that HepLPCs and BecLPCs continued to express hepatobiliary lineage genes. In contrast, the LPCs derived from the resident LPCs express neither hepatocyte-lineage markers including *Cyp1a2, Hnf1a*, nor bile duct-associated gene, Epcam, with isolated hepatocytes and BECs included as controls (Fig. 2a, Additional file 1: Fig. S2a, b). Among these markers, the expression of HNF1α and EpCAM in the three LPCs was validated by FACS analysis (Fig. 2b, Additional file 1: Fig. S1f). Furthermore, GESA analyses showed that genes of the Wnt pathways were enriched in HepLPCs and genes of the Notch pathways were enriched in BecLPCs, respectively (Additional file 1: Fig.S2c), confirming that they were independently derived from hepatic and biliary lineage.

Next, we investigated the capacity of the three LPCs for differentiation into hepatobiliary lineages in vitro. A matrigel-based 3D culture system was used to induce the formation of duct-like-organoids from three LPCs. Only BecLPCs successfully formed cysts, evidenced by uniformly higher expression of EpCAM and CK19 in BecLPC-derived cysts (Fig. 2c–e, Additional file 1: Fig. S3a, b). These findings suggested that BecLPCs were more biased toward a ductal lineage. Furthermore, 3D spheroids were generated from three LPCs to determine their hepatic differentiation capacity (Fig. 2f). After 7 days of culturing, HepLPC-derived spheroids had accumulated more glycogen and lipid and showed higher expression levels of CYP3A4 and ALB than the other two LPCs-derived spheroids (Fig. 2g, h, Additional file 1: Fig. S3c, d). Thus, HepLPC-derived spheroids preferentially displayed critical functional characteristics of hepatocytes. To further evaluate their abilities of hepatic differentiation in vivo, these differentiated LPCs (1 × 10^6^) were transplanted into *Fah*^*−/−*^ mice [17] (Fig. 2i), and all mice were sacrificed on day 30. Immunofluorescence staining showed that only HepLPCs were able to repopulate and undergo maturation in vivo due to their better hepatic functions (Fig. 2i, j). Collectively, all 3 cultured LPCs could be induced to upregulate the expression of hepatobiliary genes upon differentiation. Preferential differentiation toward hepatic and biliary lineages was observed in HepLPCs and BecLPCs, respectively, while the differentiation ability of resident LPCs-derived LPCs towards either lineage was much lower than that of HepLPCs or BecLPCs.

### The expression profile of the *in-vitro* cultured *Cd24a*^+^*Lcn2*^+^ LPCs derived from the resident LPCs recapitulated their in vivo counterparts found in ductular reactions

**Fig. 3:**
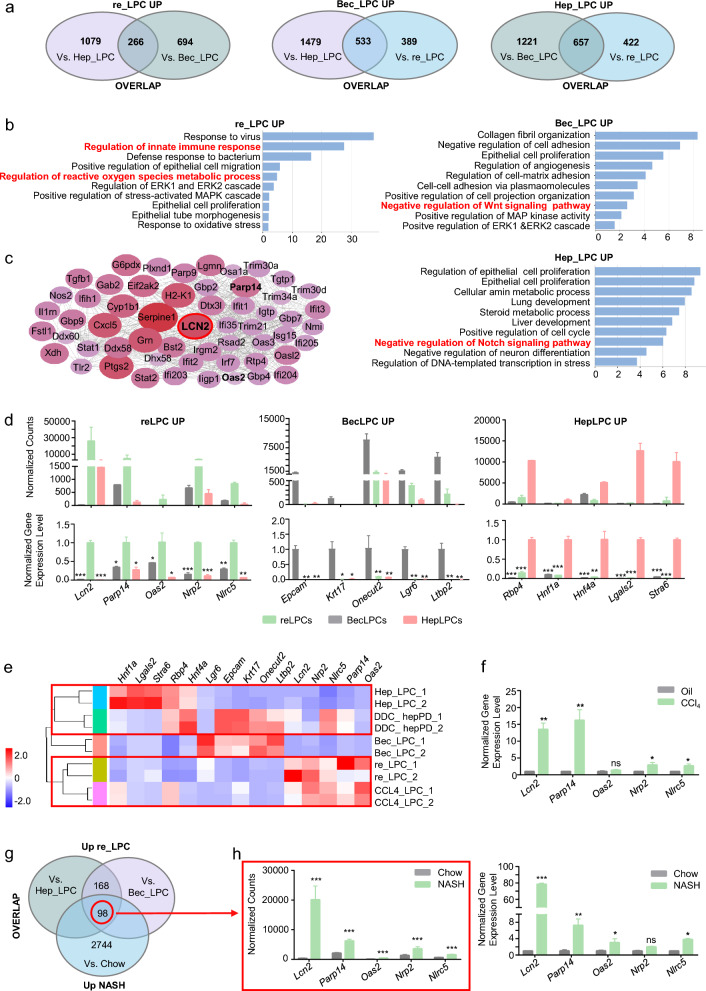
Specific markers of resident LPCs- and parenchymal cells-derived LPCs by transcriptomic profiling. **a** Venn diagrams showing numbers and overlap of genes upregulated in three cultured LPCs. **b** Ten upregulated gene ontologies (biological process) in three cultured LPCs. **c** Protein–protein interactions of DEG products in resident LPCs-derived LPCs related to gene ontologies **b** were constructed using the STRING database. The size and color of the circles depict the average absolute expression level. **d** Normalized gene counts of the 5 most significantly differentially expressed genes from RNA-sequencing **a** and validation by RT-q-PCR. Gene expression levels were normalized to Actin level and data were plotted as mean ± SD of three independent experiments. **e** Heat map showing expression levels of specific markers in three cultured LPCs, DDC-hepPD, and CCl_4_-CD24 + LPCs. **f** Normalized expression levels of 5 most significantly differentially expressed genes of resident LPCs-derived LPCs in fibrotic mice after CCl_4_ injections. **g** Venn diagrams showing numbers and overlap of genes uniquely upregulated in resident LPCs-derived LPCs by comparison with the NASH mice fed with FFC diet. **h** Normalized gene counts of 5 most significantly differentially expressed genes of resident LPCs-derived LPCs in RNA-sequencing **g** and their validation by RT-q-PCR. Gene expression levels were normalized to Actin and data are plotted as mean ± SD of three independent experiments. Resident LPCs-derived LPCs, reLPCs; Hepatocytes-derived LPCs, HepLPCs; BECs-derived LPCs, BecLPCs. For panels **d**, **f**, and **h**, data summarize 3 independent experiments. *p < 0.05; **p < 0.005; ***p < 0.001; ns: no significant difference.

Transcriptomic profiles of three cultured LPCs were generated and differential gene expression analyses were conducted to identify specific markers. In total, 533 genes were found to be BecLPC-specific, 657 HepLPC-specific, and 266 resident LPC-specific (Fig. 3a, Additional file 1: Fig. S4a, b). GO and KEGG analyses illuminated some functional differences. Genes differentially expressed by BecLPCs were enriched in negative regulation of Wnt signaling and those differentially expressed by HepLPCs concerned P450 metabolism and negative regulation of Notch signaling (Fig. 3b, Additional file 1: Fig. S4c). From each specific genes set, we found *Epcam*, *Krt17* [26], *Lgr6*, *Ltbp2*, and *Onecut2* [27] were upregulated in BecLPCs, while *Rbp4* [28], *Hnf1a, Hnf4a*, Lgals2, and Stra6 were upregulated in HepLPCs (Fig. 3d). Epithelial cell proliferation was enriched in all three LPCs, however, it is of note that innate immune and inflammation pathways, HIF-1, and oxidative stress and metabolic process were closely associated with resident LPCs (Fig. 3b, Additional file 1: Fig. S4c). In addition, GSEA validated a significant enrichment of HIF-1, IL17, JAK/STAT, and NF-kB pathways in these LPCs (Fig.S2d). Using the STRING database, we determined that LCN2 [29] was highly expressed and deeply associated with other immune and inflammatory-related genes from these *in-vitro* cultured resident LPCs specific gene sets, especially including *Parp14* [30], *Oas2* [31], *Nrp2* [32], and *Nlrc5* [33] (Fig. 3c, d).

Based on these potential markers groups for discrimination of LPC source, we compared the expression profile of hepatocyte-derived proliferative ducts (hepPDs) from animals receiving DDC diet [7] with that of cultured LPCs (Fig. 3e). Heatmap generated from the expression of specific markers showed that the expression level of these markers in DDC-hepPDs was comparable to that of cultured LPCs. Furthermore, we observed that DDC-hepPDs displayed a closer clustering with HepLPCs rather than *Cd24a*^+^*Lcn2*^+^ LPCs or BecLPCs, suggesting the correlation between the in vitro-cultured LPCs and the state of their in vivo counterparts. In accordance with this correlation, we also compared the expression profile of isolated *Cd24a*^+^ LPCs after CCl_4_ treatment in vivo (CCl_4_-LPC) [21] with that of *in-vitro* cultured *Cd24a*^+^*Lcn2*^+^ LPCs (Fig. 3e). Consistently, the heatmap showed that CCl_4_-LPCs were more closely interrelated to *Cd24a*^+^*Lcn2*^+^ LPCs. These results were further confirmed by quantitative PCR analysis of the specific markers in CCl_4_-treated and normal groups (Fig. 3f). These findings suggested that endogenous *Cd24a*^+^*Lcn2*^+^LPCs in hepatic ductular reactions might originate from the pre-existing resident LPCs.

### Single-cell RNA sequencing and spatial localization of resident LPCs-derived *Cd24a*^+^*Lcn2*^+^ LPCs

**Fig. 4:**
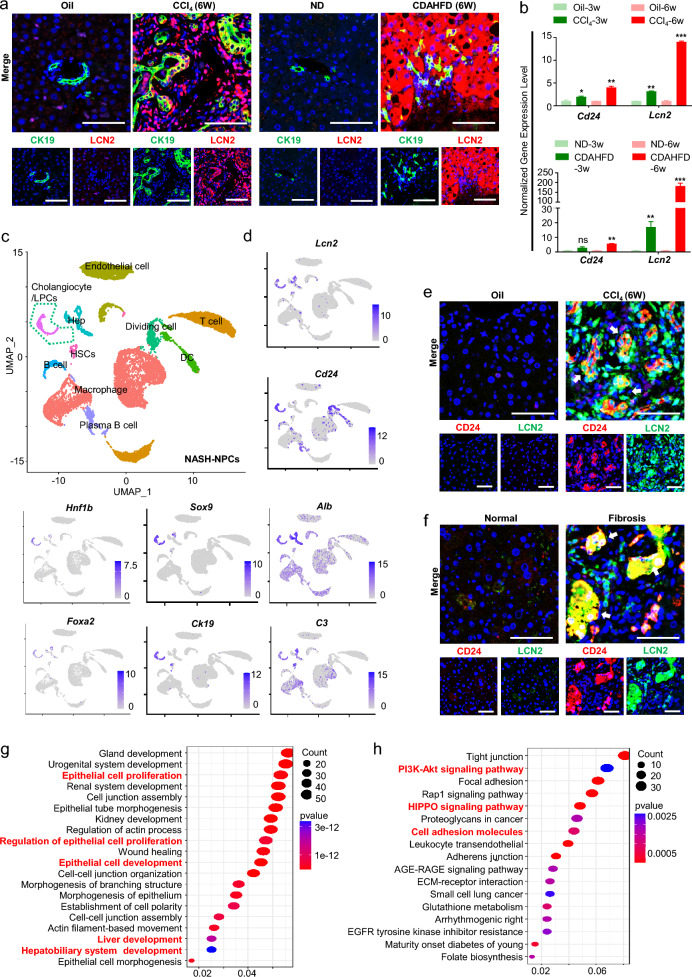
Single-cell RNA sequencing and spatial location of resident LPCs-derived *Cd24a*^+^*Lcn2*^+^ LPCs activated in chronic liver disease models. **a** Representative immunofluorescence staining of LCN2 and CD24 expression in different hepatic disease models. CDAHFD, Choline-Deficient L-Amino Acid-Defined High-Fat Diet; ND, normal diet. Scale bar: 100 μm. **b** RT-q-PCR analysis of *Lcn2* and *Cd24* expression in mice's fibrotic and NASH liver at different time points. The data are expressed as means ± SD of three independent experiments. *p < 0.05; **p < 0.005; ***p < 0.001. **c** Liver NPCs were isolated from the livers of mice fed with the AMLN diet to induce NASH and subjected to scRNA-Seq. U-map visualization of liver cell clusters based on 15,380 single-cell transcriptomes. **d** U-map of NASH-NPCs. The feature plots show *Lcn2*, *Cd24a*, *Hnf1b*, *Foxa2*, *Ck19*, *Sox9*, *Alb* and *C3* expression levels. The color bar indicates the expression level of scaled genes. **e** Representative immunofluorescence staining of LCN2 and CD24 expression in mouse fibrotic liver treated with CCl_4_. Scale bar: 100 μm. **f** Representative immunofluorescence staining of LCN2 and CD24 expression in human fibrotic liver tissues. Scale bar: 100 μm. **g** GO enrichment pathway analysis of *Cd24a*^+^*Lcn2*^+^ LPCs clusters in NASH-NPCs. **h** KEGG enrichment pathway analysis of Cd24a^+^Lcn2^+^ LPCs clusters in NASH-NPCs

The degree of ductal reactions was positively correlated with disease severity from a range of pathogenic causes like NASH [34, 35]. *Cd24a*^+^*Lcn2*^+^ LPC-specific genes overlapped with genes differentially expressed in mice fed a diet rich in fat, fructose, and cholesterol (FFC) to cause NASH (Fig. 3g, h). Notably, LCN2 was also shown to be upregulated in ductular reactive cells during CCl_4_-induced liver fibrosis model and in the NASH model induced by Choline-Deficient L-Amino Acid-Defined High-Fat Diet (CDAHFD) [36, 37] (Fig. 4a). In addition, these models with more severe hepatic injury showed a higher expression of LCN2 and CD24 by qPCR, especially in NASH models (Fig. 4b).

To further clarify the identity of these *Cd24a*^+^*Lcn2*^+^ LPCs, we analyzed the single-cell RNA sequencing data of NASH-induced NPCs [38]. Although Cd24a was expressed in different immune cells (macrophages, DCs, and B cells) while LCN2 in hepatocytes, a subpopulation of LCN2 and Cd24a positive cells was identified with not only abundant stem/progenitor markers of *Sox9, Hnf1b,* and *Foxa2* but also hepatobiliary functional genes such as *Alb/C3/Krt19* (Fig. 4c, d). Immunofluorescence staining defined the cellular morphology and spatial location of these endogenous *Cd24a*^+^*Lcn2*^+^ LPCs in regions of ductular reaction (Fig. 4e). Analysis of clinical samples from patients with liver fibrosis also showed a huge abundance of endogenous CD24^+^LCN2^+^ cells in ductular reaction foci (Fig. 4f, Additional file 1: Fig. S7a). GO and KEGG analyses of the *Cd24a*^+^*Lcn2*^+^ subset showed they were mostly enriched in epithelial cell proliferation, hepatobiliary system development, and Hippo signaling pathway (Fig. 4g, h). These findings suggested that *Cd24a*^+^*Lcn2*^+^ exhibited an LPCs-like profile and contributed to ductular reactions in chronic liver diseases. Hence, Cd*24a* and *Lcn2* could be jointly utilized to identify the subset of LPC that mainly originated from resident LPCs.

### CD24^+^LCN2^+^ LPCs elicit a robust macrophage response in vitro and in vivo

**Fig. 5:**
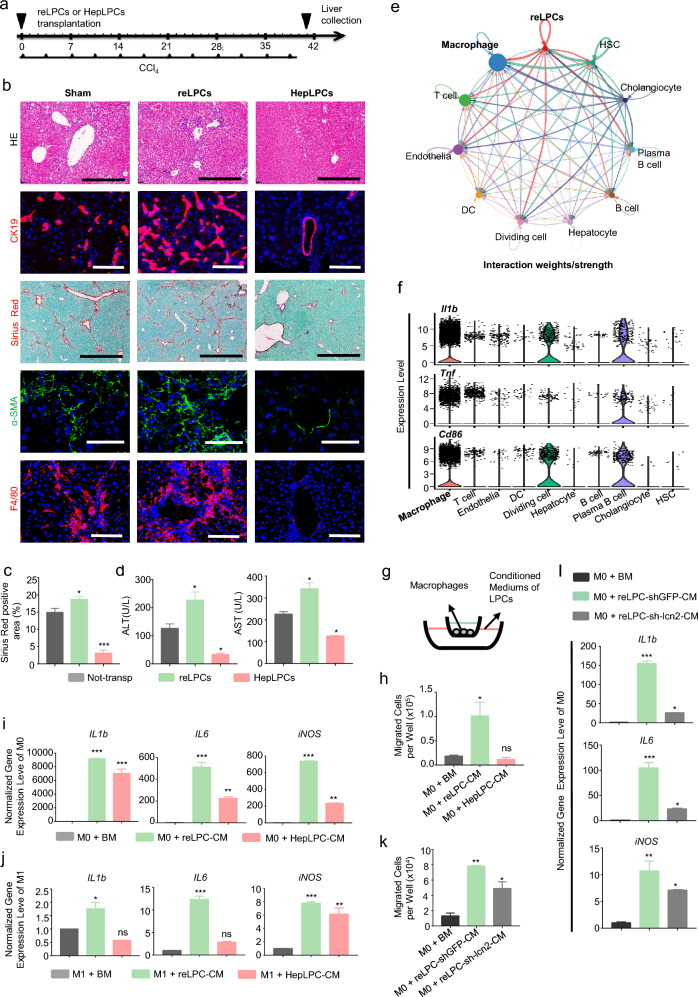
Transplantation of CD24^+^LCN2^+^ LPCs exacerbated chronic fibrosis and inflammation by infiltrating macrophages. **a** Schematic overview of the experimental design. Eight-week-old wild-type C57BL/6 mice were injected with 2 mL/kg CCl_4_ i.p. twice a week for a continuous period of 6 weeks. 1–2 × 10^6^ CD24^+^LCN2^+^ LPCs or HepLPCs were transplanted 4 h after the first CCl_4_ injection. **b** Representative images of H&E, Sirius Red, and immunofluorescence staining for CK19, α-SMA, and F4/80 in liver slides. Scale bar: 600 μm for H&E staining and Sirius Red staining; 200 μm for immunofluorescence staining. **c** Quantification of Sirius Red positive area as a proportion of the total liver area. Data were measured by Image J software. **d** Serum analysis of ALT and AST. **e** Circle plots showing the number of interactions between CD24^+^LCN2^+^ LPCs and other cell groups in NASH-NPCs of Fig. 4. **f** Violin plots showing expression of Il1b, Tnf, and Cd86 in NPCs of Fig. 4. **g** Schematic model of the transwell assay and quantification of migrated cells in basal medium, CD24^+^LCN2^+^ LPC conditioned medium or HepLPC conditioned medium. **h** Quantification of the cell number at the bottom of transwells. **i** RT-q-PCR analyses M1 macrophage expression. M0 macrophages were cultured in basal medium (BM) or conditioned media (CM) of CD24^+^LCN2^+^ LPCs or HepLPCs, as indicated. **j** RT-q-PCR analyses of M1 macrophage expression. M1 macrophages were cultured in basal medium (BM) or conditioned media (CM) of CD24^+^LCN2^+^ LPCs or HepLPCs, as indicated. **k**, **l** Quantification of the cell number at the bottom of transwells **k** and RT-q-PCR analyses of M1 macrophage expression **l** M0 macrophages were cultured in basal medium (BM) or CD24^+^LCN2^+^ LPC-conditioned mediums with or without Lcn2 expression. Resident LPCs-derived CD24^+^LCN2^+^ LPCs, reLPCs; Hepatocytes-derived LPCs, HepLPCs. For panels c, d, h, i, j, k, and l, data are expressed as means ± SD of three independent experiments. p values were analyzed by one-way ANOVA. *p < 0.05; **p < 0.005; ***p < 0.001; ns: no significant difference

As shown in revised Fig. 4a, b, we noted more LCN2-positive hepatocytes in CDAHFD-fed mice than in CCl4-treated mice. These excessively high levels of hepatocytes-derived LCN2 in NASH model might hamper investigating the roles of LPCs-derived LCN2 against hepatic fibrosis as compared to the CCl_4_ model. We, therefore, determined to transplant GFP/Luciferase labeled CD24^+^LCN2^+^ LPCs (2 × 10^6^) into CCl_4_-treated mice (Fig. 5a, Additional file 1: Fig. S5a), and HepLPCs were considered as controls. Both CD24^+^LCN2^+^ LPCs and HepLPCs could be found by live imaging 24 h after transplantation (Additional file 1: Fig. S5b). Although HepLPCs transplantation reduced liver injury as reported before [21], CD24^+^LCN2^+^ LPC transplantation enhanced ductular reaction and liver fibrosis in H&E, Sirius Red, CK19, and α-SMA staining, and increased the serum levels of ALT and AST compared to control mice on day 40 (Fig. 5b–d, Additional file 1: Fig. S5c, d). These results indicate that CD24^+^LCN2^+^ LPCs exacerbated, rather than ameliorate, liver damage.

LCN2 recruits inflammatory cells and triggers inflammatory response [39]. Consistently, transplantation of CD24^+^LCN2^+^ LPCs with high expression of *Lcn2* enhanced the tissue infiltration of F4/80^+^ macrophages and neutrophils, as shown by MPO staining (Fig. 5b, Additional file 1: Fig. S5c, e). The CellChat toolkit of sc-RNA seq was used to investigate the cell–cell interaction network between *Cd24a*^+^*Lcn2*^+^ cells and other NPCs in Fig. 4c [40, 41], showing *Cd24a*^+^*Lcn2*^+^ cells had the strongest interaction with M1 macrophages [42] (Fig. 5e, f). In vitro transwell assays demonstrated that CD24^+^LCN2^+^ LPCs exerted an enhanced chemotactic effect on bone marrow-derived macrophages (BMDMs, Fig. 5g, h). Moreover, BMDMs cultured with CD24^+^LCN2^+^ LPC conditioned medium expressed higher levels of M1 markers, including IL-1b, IL-6, and inducible nitric oxide synthase (iNOS; Fig. 5i, j, Additional file 1: Fig. S6a, b), while BMDMs cultured with HepLPC conditioned medium expressed higher levels of M2 marker, ARG1 (Additional file 1: Fig. S6c). Silencing of the *Lcn2* gene in CD24^+^LCN2^+^ LPCs eliminated the chemotactic paracrine action on macrophages and prevented up-regulation of M1 marker genes (Fig. 5k, l, Additional file 1: Fig. S6d, e). In summary, replenishment of CD24^+^LCN2^+^ LPCs provoked an enhanced macrophage response, which might exacerbate chronic liver damage.

### Single-cell atlas revealed *CD24*^+^*LCN2*^+^ LPC-macrophage cell–cell communication network in human cirrhotic liver

**Fig. 6:**
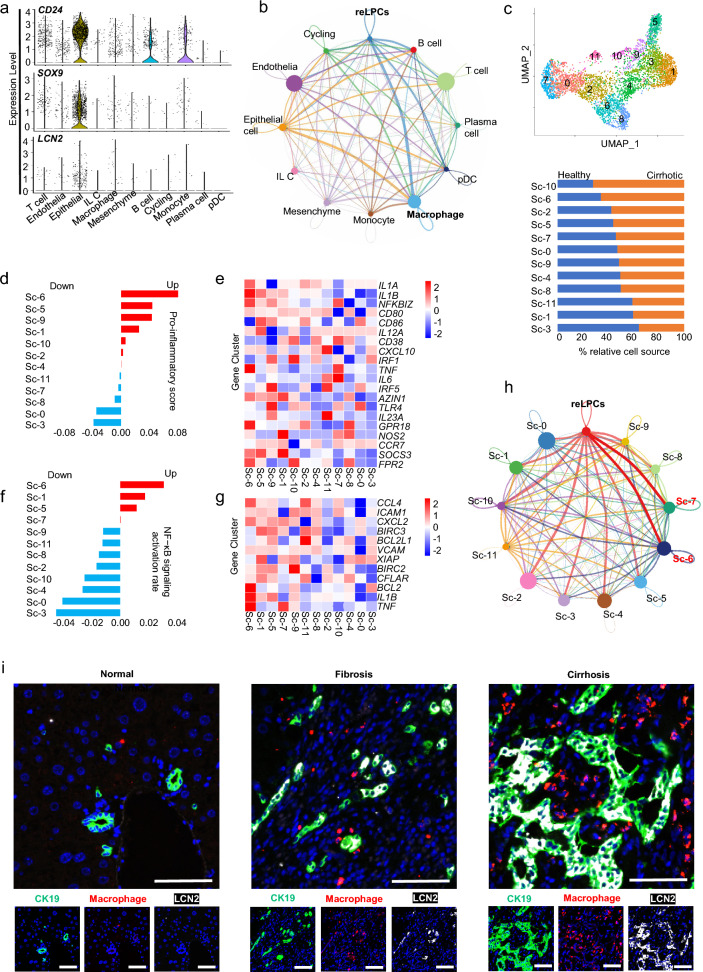
Single-cell atlas validated *CD24*^+^*LCN2*^+^ LPC-macrophage cell–cell communication network in human cirrhotic liver disease. **a** Violin plots showing expression of LCN2, CD24, and SOX9 in NPCs from epithelia of the human cirrhotic liver. **b** Cellchat showed the number of interactions between *CD24*^+^*LCN2*^+^ LPCs and other cell groups in human liver NPCs diagnosed with cirrhosis. **c** U-map visualization of macrophage clusters is based on 4648 single-cell transcriptomes and the ratio of the macrophages of different clusters to total macrophages between cirrhotic and healthy livers. **d**, **e** The heatmap and the score of different pro-inflammatory markers in different clusters of Kupffer cells are based on the analysis by QuSAGE (2.28.0), with the gene set shown in the heatmap. **f** The activation rate of the NF-kB signal pathway in different clusters is based on the analysis by QuSAGE (2.28.0) of the gene set from the KEGG database. **g** The heatmap of NF-kB target genes in different clusters of macrophages. **h** Cellchat showed the number of interactions between *CD24*^+^*LCN2*^+^ LPCs and other cell groups of macrophages in cirrhotic liver NPCs. **i** Representative immunofluorescence staining of CK19, LCN2, and F4/80 expression in clinical samples of liver fibrosis and cirrhosis. Scale bar: 200 μm. Resident LPCs-derived CD24^+^LCN2^+^ LPCs, reLPCs

The dataset of NPC types in cirrhotic liver disease was partitioned into clusters and annotated using signatures of known lineage markers (Additional file 1: Fig.S7b). The cell subset with high expression of *CD24, LCN2,* and *SOX9* was associated with epithelia (Fig. 6a) and showed especially strong interactions with macrophages (Fig. 6b). Unsupervised U-map analysis identified 12 sub-clusters (sc-0–11) of macrophages, among which sc-10/6/2/5/7 was the most enriched in cirrhotic livers (Fig. 6c). Single-cell transcriptome analysis revealed that sc-6 had the highest upregulation of pro-inflammatory genes and proinflammatory surface markers than other sub-clusters (Fig. 6d, e). The quantitative analysis of NF-kB target genes revealed that sc-6/1/5/7 scored higher among all 12 sub-clusters (Fig. 6f, g). The sc-6 and sc-7 subpopulations had enhanced pro-inflammatory phenotypes with the strongest interactions with *CD24*^+^*LCN2*^+^ LPCs (Fig. 6h).

Finally, as ductular reaction aggravated with the progression of fibrosis to cirrhosis, immunofluorescent staining showed increasing numbers of LCN2^+^ LPCs surrounded by more macrophages in liver tissues (Fig. 6i, Additional file 1: Fig. S7c). Thus, the single-cell atlas of human liver NPCs and immunofluorescent staining validated the involvement of CD*24*^+^*LCN2*^+^ LPCs in ductular reactions, contributing to the exacerbation of inflammation and fibrosis by macrophage recruitment.

## Discussion

Whether LPCs of different origins contribute to injury-induced liver regeneration and the underlying mechanisms have long been debated. We previously reported a small-molecule cocktail for in vitro maintenance and expansion of HepLPCs [17, 18] and have cultured LPCs from the resident LPCs and BECs in this study. Transcriptomic and functional analyses have shown that LPCs of different origins shared common progenitor cell features, such as increased expression of proliferative markers (*Cd24 and Sox9*), and enrichment of stemness-related pathways.

Additionally, transcriptomic and functional comparisons of cultured LPCs revealed specific markers for each cell type of LPCs and illuminated their functional differences in the treatment of liver injury. LCN2 was identified as a specific marker of the activated resident LPCs, distinguishing it from HepLPCs and BecLPCs. Using the STRING database, we determined that LCN2 was highly expressed and deeply involved in innate immunity and inflammation, IL-17 signaling pathway, and cellular response to reactive oxygen species. LCN2 is a secretory glycoprotein belonging to the lipocalin superfamily but is lowly expressed in healthy adults [43]. The upregulation of LCN2 expression in damaged hepatocytes and infiltrating immune cells has been observed in various liver diseases, including hepatitis, alcoholic liver disease, and non-alcoholic steatohepatitis [44, 45]. Correspondingly, animal models treated with bile duct ligation, repeated CCl_4_ injections, and CDAHFD diet, also upregulate LCN2 in damaged livers, resulting in aggravating the inflammatory response [39]. Interestingly, LCN2 had higher expression in the CDAHFD diet-induced NASH model than in the CCl_4_-induced liver fibrosis model according to our PCR data. There might be several reasons for this observation. First, mice receiving corn oil had higher levels of LCN2 than mice receiving a normal diet [44]. Second, a higher degree of damage (including steatosis and fibrosis) occurred in mice receiving 6 weeks of CDAHFD diet than in mice receiving 6 weeks of CCl_4_. In addition, compared with WT mice with a high-fat diet (HFD) to induce simple steatosis, HFD-fed ob/ob mice (models of NASH) had higher hepatic expression of LCN2 and larger Sirius Red-stained fibrotic areas [46]. This study demonstrated that LCN2 level was correlated with the severity of liver inflammation and the stage of hepatic fibrosis, making it a good candidate biomarker or even a therapeutic target for NAFLD and fibrosis [45, 46].

The transcriptional profile of cultured CD24^+^LCN2^+^ LPCs was comparable to that of endogenous CD24^+^ LPCs in ductular reaction foci [21]. Furthermore, the presence of CD24^+^LCN2^+^ LPCs was confirmed by sc-RNA seq data of NPCs and immunofluorescent staining of chronic liver injury models. These findings may recapitulate the cellular plasticity found in ductular reactions in vivo and support the hypothesis that the resident progenitor/stem cells derived LPCs may drive ductular reaction [2]. A subpopulation of LCN2-expressing CD24^+^ LPCs was activated and expanded in ductular reaction foci with liver disease progressed. LPCs have been reported to secrete chemokines and proinflammatory mediators during ductular reactions, promoting macrophage and neutrophil infiltration into the periportal area [47, 48]. Upregulation of LCN2 had a hepatoprotective effect in acute liver injury but accelerated the development of chronic cirrhosis and facilitated the crosstalk between neutrophils and Kuppfer cells, thereby worsening steatohepatitis [29]. In addition, LCN2 acts as a key mediator of HSC activation in leptin-deficient obesity via α-SMA/MMP9/STAT3 signaling, further exacerbating NASH [46]. In this study, cultured CD24^+^LCN2^+^ LPCs elicited robust inflammatory responses from macrophages in an LCN2-dependent manner, and transplantation of CD24^+^LCN2^+^ LPCs into CCl_4_-treated mice promoted macrophage infiltration and induced M1 polarization. We hypothesized that silencing LCN2 in CD24^+^LCN2^+^ LPCs might alleviate hepatic damage, since depletion of LCN2 substantially attenuated necroinflammation and infiltration of neutrophils and macrophages, protecting against HFD-induced steatohepatitis and fibrosis [29]. Thus, the current study has established the role of CD24^+^LCN2^+^ LPCs in the inflammatory response of macrophage in ductular reactions responses, worsening hepatic dysfunction.

We acknowledge some limitations to the current study. Firstly, consistent with the small-molecule-induced expansion of LPCs in vitro [24], we speculated that CD24^+^LCN2^+^ LPCs were derived from the resident LPCs. In addition, LCN2 was identified as a critical marker and the proinflammatory effects to distinguish resident LPCs-derived LPCs from other types of LPCs. However, it is unknown whether LCN2 exists in the resident LPCs in healthy humans or whether its silencing in CD24^+^LCN2^+^ LPCs promotes liver regeneration. Secondly, in vitro spheroid and organoid formation assays demonstrated that all three LPCs could be induced to upregulate hepatobiliary gene expression upon differentiation, however, spontaneous and preferential differentiation did exist in distinct LPCs. Further studies are needed to explore whether a modified medium with factors like wnt3a, Rspo1, and NOGGIN [49–51] can enhance the differentiation potency of BecLPCs and CD24^+^LCN2^+^ LPCs toward the hepatic lineage. Finally, in this study, we proved that a large amount of CD24^+^LCN2^+^ LPCs were activated during chronic liver diseases and contributed to exacerbating hepatic damage. However, we could not rule out the existence of transitional liver progenitor cells (TLPCs), which originates from BECs during regeneration [52]. Meanwhile, further studies are necessary to establish whether CD24^+^LCN2^+^ LPCs, a major LPC type in ductular reaction, have a ‘reprogramming competence’ to reduce hepatocyte and biliary epithelial cell plasticity (like hepPDs and TLPC) during liver fibrosis and NASH.

## Conclusion

In summary, CD24^+^LCN2^+^ LPCs were activated during chronic liver disease, contributed to macrophage infiltration and polarization, and exacerbated liver inflammation and fibrosis. These findings illuminated the distinct roles of LPCs of different origins, which may aid the identification of appropriate LPCs for treating chronic hepatic diseases.

## Materials and methods

### Cell isolation, culture, and fluorescence-activated cell sorting (FACS)

Aliquots of 2 × 10^11^ plaque-forming units (pfu) of adeno-associated virus (AAV) vector containing Cre recombinase regulated by the thyroxine-binding globulin promoter (AAV8-*Tbg-Cre*, Celliver Biotechnology Inc., Shanghai, China) were intravenously injected into 8-week-old *C57BL/6-Gt (ROSA26)*^*tm1(CAG−LSL−Tdtomato)*^*/Bcgen* mice (*ROSA26*^*tdTomato*^, the Jackson Laboratory). tdTom-negative hepatocytes (tdTom^−^) were converted into tdTom-positive (tdTom^+^) after 14 days. When foregut endoderm cells were ready to express the liver-specific gene, *Alb* [5], *Alb*^*CreERT*^*/R26*^*GFP*^ mice bred from B6.129S-Albtm1.1(CreERT2) Smoc (*Alb*^*CreERT*^) and *C57BL/6-Gt (ROSA26) *^*Sortm1(CAG−DTR−EGFP)*^*/Bcgen* (*ROSA26*^*DTR−EGFP*^) were used for genetic labeling of albumin (ALB)-expressing cells and characterized by genotyping. These tdTom-positive (tdTom^+^) hepatocytes and ALB and EpCAM-negative (GFP^−^EpCAM^−^) cells were isolated using a two-step collagenase perfusion protocol, as described previously [21].

BECs were isolated from 6 to 8 week old mice as described [53]. Using the dissecting microscope, hepatocytes, portal vein branches, and connective tissues were removed after liver perfusion. Pieces of bile ducts were immersed in a solution of digestive enzymes for 20 min (Celliver Biotechnology Inc.). Then, small pieces of bile ducts were incubated into matrigel and cultured in Transition and Expansion Medium (TEM). After 1–2 passages, cholangiocytes grew out of the pieces and formed a monolayer. Single-cell suspensions were sorted by Beckman MoFlo XDP equipped with 405 nm, 488 nm, 561 nm, and 640 nm excitation lasers.

Three types of FACS-sorted cells were cultured in TEM, as described previously [17]. Briefly, TEM was based on DMEM/F12 (Invitrogen) supplemented with N2 and B27 (Invitrogen) and the following growth factors or small molecules: 20 ng/mL EGF, 20 ng/mL HGF (all Peprotech), 10 μM Y27632, 3 μM CHIR99021, 1 μM S1P, 5 μM LPA, and 1 μM A83-01 (all TargetMol). All mouse experiments were performed in accordance with the Guide for the Care and Use of Laboratory Animals. The Institutional Animal Care and Use Committee at the Shanghai Model Organisms Center Inc. approved this study.

### RNA sequencing and bioinformatics analysis

Total RNA was isolated using the RNeasy mini kit (Qiagen, Germany), quantified by NanoDrop ND-2000 spectrophotometer (Thermo Fisher Scientific, Waltham, MA, USA) and integrity was determined by the Agilent 2100 system and RNA 6000 Nano kit (Agilent Technologies, Santa Clara, CA, USA). Paired-end libraries were constructed using TruSeq Stranded mRNA LTSample Prep Kit (Illumina, San Diego, CA, USA), according to the manufacturer's instructions. Libraries were sequenced on an Illumina platform (HiSeq X Ten, Illumina, Shanghai OE Biotech. Co., Ltd.), and 150 bp paired-end reads were generated. Fastp software (v0.20.0) was used to trim adaptor and remove low-quality reads to get high-quality clean reads. STAR software (v2.7.9a) was used to align high-quality clean reads to the reference genome. featureCounts software (v2.0) was used to get the raw gene level mRNA expression counts. Original data were uploaded to the Gene Expression Omnibus database (GSE135951 and GSE125095). Other data were downloaded from GEO as follows: hepatocytes and BECs: GEO Accession No. GSE156894; Macrophages: GEO Accession No. GSE152211; Hepatic stellate cells: GEO Accession No. GSE96526; liver sinusoidal endothelial cells: GEO Accession No. GSE164006; Hepatocyte-derived proliferative ducts (hepPDs): GEO Accession No. GSE55552 and Chow and FFC-induced NASH mice: GEO Accession No. GSE164084.

Single-cell RNA analysis was conducted by collating single-cell expression data from NPCs isolated from CCl_4_-induced liver fibrosis model (GEO Accession No. GSM5548830), non-alcoholic steatohepatitis (NASH) models (GEO Accession No. GSM3714750, GSM3714751, GSM3714752) and patients with cirrhosis (GEO Accession No. GSM4041161-GSM4041169). A total of 15,380 cells from NASH-NPCs, 4697 cells from CCl_4_-NPCs, and 25,477 cells from human cirrhosis-NPCs passed the quality control threshold of > 500 transcripts. Any genes detected in fewer than three cells (UMI count > 0) were removed. All datasets were normalized using log_2_CPM and the raw count matrix (UMI counts/gene/cell) was processed by Seurat.

Sample clustering and soft threshold screening were conducted using a Euclidean distance metric with complete linkage [54]. GSEA analysis (GSEA, Broad Institute) was used to classify upregulation by fold change and genes [55]. Gene Ontology (GO), Kyoto Encyclopedia of Genes and Genomes (KEGG), Cellchat, and QuSAGE (2.28.0) [56] analysis were performed. Data were analyzed using R software based on the hypergeometric distribution.

### Quantification and statistical analysis

Samples were randomly collected from five mice per condition and from five independent liver fields per mouse. Statistical analysis was performed using GraphPad Prism 7. A two-tail unpaired t-test was used to compare means, and one-way ANOVA with Dunnett correction was used for multiple comparisons with single variables. A *p*-value < 0.05 was considered statistically significant.

More details on materials and methods are provided in the Supplementary File.

### Supplementary Information

**Additional file 1.** Additional methods, figures and tables.

## Data Availability

The datasets generated during the current study will be available from the corresponding author upon reasonable request.
