# Supplementary material for: CD24+LCN2+ liver progenitor cells in ductular reaction contributed to macrophage inflammatory responses in chronic liver injury
Source: Cell Biosci. 2023 Oct 2;13:184. doi: 10.1186/s13578-023-01123-2 (PMC10546777; doi:10.1186/s13578-023-01123-2)
Supplement: Supplementary file 1 — Additional file 1. Additional methods, figures and tables. [file 13578_2023_1123_MOESM1_ESM.docx]

**Additional file 1 Materials for a manuscript by Huang *et. al.***

Wei-Jian Huang^1,2,3,*^, Bi-Jun Qiu^4,*^, Xiao-Shu Qi^1,2,*^, Cai-Yang Chen^1,*^, Wen-Ming Liu^1,2,5^, Shen-ao Zhou^3^, Min Ding^6^, Feng-Feng Lu^5^, Jie Zhao^4^, Dan Tang^1,2^, Xu Zhou^1,2^, Gong-Bo Fu^7^, Zhen-Yu Wang^8^, Hong-Qian Ma^1,2^, Yu-Ling Wu^1,2^, Hong-ping Wu^9^, Xiao-Song Chen^10,#^, Wei-Feng Yu^1,2,#^ and He-Xin Yan^1,2,3,6,8,#^

**Author affiliations**

From the ^1^Department of Anesthesiology and Critical Care Medicine, School of Medicine, Renji Hospital, Shanghai Jiaotong University, Shanghai, China; ^2^Key Laboratory of Anesthesiology (Shanghai Jiao Tong University), Ministry of Education, China; ^3^Celliver Biotechnology Inc., Shanghai, China; ^4^Department of Liver Surgery, School of Medicine, Renji Hospital, Shanghai Jiao Tong University; ^5^Institute for Regenerative Medicine, Shanghai East Hospital, School of Life Sciences and Technology, Tongji University, Shanghai, China; ^6^Department of Interventional Oncology, School of Medicine, Renji Hospital, Jiaotong University, Shanghai, China; ^7^Department of Medical Oncology, Jinling Hospital, First School of Clinical Medicine, Southern Medical University, Nanjing, China; ^8^State Key Laboratory of Oncogenes and Related Genes, Shanghai Cancer Institute, Renji Hospital, School of Medicine, Shanghai Jiaotong University, Shanghai, China; ^9^International Cooperation Laboratory on Signal Transduction, Eastern Hepatobiliary Surgery Hospital, Second Military Medical University, Shanghai, China; ^10^Department of Infectious Diseases, Renji Hospital, Shanghai Jiaotong University School of Medicine, Shanghai, China.

**Table of Contents**

| Additional file 1 Methods | Page 3 |
| --- | --- |
| Additional file 1 Figure 1 | Page 8 |
| Additional file 1 Figure 2 | Page 10 |
| Additional file 1 Figure 3 | Page 12 |
| Additional file 1 Figure 4 | Page 13 |
| Additional file 1 Figure 5 | Page 15 |
| Additional file 1 Figure 6 | Page 16 |
| Additional file 1 Figure 7 | Page 18 |
| Additional file 1 Table 1 | Page 19 |
| Additional file 1 Table 2 | Page 21 |
| Additional file 1 Table 3  Additional file 1 Table 4  Additional file 1 Table 5 | Page 23  Page 25  Page 26 |
| References | Page 27 |

**Additional file 1 methods**

**Mouse experiments**

Housing, husbandry, and all experimental protocols for mice used in this study were performed following the guidelines established by the Institutional Animal Care and Use Committee at the Shanghai Model Organisms Center Inc. All animals were housed in individually ventilated cages (IVC) with temperature (22-25°C) and light (12 h light/dark cycle) controlled specific pathogen-free (SPF) animal facility, and had ad libitum access to food and water in Shanghai Model Organisms Center Inc. Eight-week-old male mice obtained from Institute of Laboratory Animal Sciences, CAMS & PUMC (Beijing, China) were used for all experiments and were randomly distributed into experimental and control groups for each study.

To induce hepatic fibrosis, mice were intraperitoneally injected with CCl_4_ of 2 mL/kg body weight (Sigma-Aldrich; CCl_4_ was dissolved in olive oil at a ratio of 1:4) for the experimental groups, or olive oil alone for the control group (2 ml/kg) twice a week for 6 weeks (1). For cell transplantation, 1-2.0×10^6^ HepLPCs or CD24^+^LCN2^+^ LPCs in 200 μL of William’s E medium were injected into hepatic fibrosis mice through the spleen using a 27-gauge needle. As for the control (non-transplantation) group, 200 μL of William’s E medium was injected in the manner mentioned above. After transplantation of cells with lentivirus vector carrying luciferase (Luc), mice were measured and quantified by *ex vivo* bioluminescent imaging at different time points using IVIS Lumina Series III (PerkinElmer, USA).

Before transplantation of LPCs into Fah^-/-^ mice, 2-(2-nitro-4-trifluoro-methylbenzyol)-1,3-cyclohexanedione (NTBC) was withdrawn. After intrasplenic transplantation of 1x10^6^ differentiated LPCs, mice were closely monitored and weighed every other day. NTBC was transiently put on for two days when mice lost 20% of their body weight and all mice were sacrificed at day 30.

**The organoid and spheroid culture**

For three-dimensional spheroid culture, 1×10^6^ LPCs were seeded on low-attachment 6-well plates, and cellular spheres were cultured in TEM/HMM (1:1) for 7-10 days(2). To culture organoids, cell pellets were resuspended with optimized organoid culture medium that was composed of advanced DMEM/F-12 medium supplemented with 1% penicillin/streptomycin, 2 mM GlutaMAX-1, 10 mM HEPES, 100 μg/mL Primocin, 2% B27 supplement (without Vitamin A), 10 mM Nicotinamide, 1.56 mM N-acetyl-l-cysteine, 50 ng/mL recombinant EGF, 10 ng/mL recombinant FGF-10, 25 ng/mL recombinant HGF, 10 µM forskolin, 500 nM A-83-01, 10 µM Y-27632. One to two thousand isolated cells were mixed with cold Matrigel Basement Membrane Matrix (Corning), and 50 μL of the cell and Matrigel mixture was placed on prewarmed 24-well suspension culture plates at 37 °C for 30 min to solidify. Upon complete gelation, 1 mL of organoid medium was added to each well, and plates were transferred to the humidified 37℃ incubators with 5% CO_2_. The culture medium was replaced every 3 to 4 days for organoid growth.

**Staining and imaging**

Paraffin-embedded tissue sections (3 μm) were deparaffinized and rehydrated with the graded ethanol concentrations. The information on primary antibodies for immunohistochemistry was listed in Additional file 1 Table 3 (Antibodies list). Sections were stained with Sirius Red/Fast Green (Chondred), antibodies, or hematoxylin&eosin (H&E) according to routine protocols. For PAS, after deparaffinization and rehydration, sections were stained by Periodic-Acid-Schiff (PAS, Sigma) following the manufacturer's instructions.

For immunofluorescence staining, liver tissues or organoids were fixed at 4°C overnight in 4% paraformaldehyde. Then the tissues were put into graded sucrose solutions and embedded in OCT (Tissue Tek). The information on incubated antibodies was listed in Additional file 1 Table 3 (Antibodies list). For Oil-red staining, OCT (Tissue Tek)-embedded sections were incubated with Oil-red (Sigma-Aldrich) according to the manufacturer's instructions. MDR1-mediated transport of Rhodamine-123 was detected over a 10-15-minute incubation as described previously(3).

Representative images of Sirius Red, H&E, and immunohistochemical staining were attained from Leica Aperio AT Turbo, while that immunofluorescence staining was collected by Leica TCS SP8. Besides, images of PAS and Oil-red staining results were acquired via Olympus IX70, and Image J software was applied to analyze the Sirius results.

**Transwell assay & *in vitro* macrophages studies**

To prepare the conditioned medium, HepLPCs or CD24^+^LCN2^+^ LPCs were cultured to 60% confluence in culture dishes. After being washed with PBS, cells were incubated in the same volume of serum-free Dulbecco’s modified Eagle’s medium (DMEM) for 48 h. The conditioned medium was harvested and centrifugated at 300 g for 5 min to remove cell debris.

For transwell assay, BMDMs were prepared and cultured as described previously(4). In brief, bone marrows were flushed from the femurs and tibias of mice and dispersed mechanically. Red blood cells were lysed using the ACK lysis buffer (Thermo), and the remaining cells were collected by centrifugation at 300 g for 5 min. After centrifugation, 2 × 10^5^ monocytes were seeded on the upper chamber of the transwell (24-well plate, Corning, 3415) filter with 3 μm pores (Falcon) or dishes in DMEM supplemented with 10% fetal bovine serum (FBS) and macrophage colony-stimulating factor (M-CSF, 20 ng/mL, Sino Biological Inc., Beijing) for 7 days. Then the bottom medium was changed to the conditioned medium of LPCs for 6 hours at 37℃, Normoxia (5% CO_2_ incubator). After culturing for 6 hours, the bottom medium was collected for quantifying the cell number by ﬂow cytometry using the absolute quantification method.

For M1 macrophage induction, BMDMs were stimulated with lipopolysaccharide (LPS, 50 ng/mL, Sigma) and IFN-γ (20 ng/mL, PeproTech, Rocky Hill, CT) for 24 h. Then M0 or M1 was treated with the conditioned medium of HepLPCs or CD24^+^LCN2^+^ LPCs for 6 hours before detecting gene expression *via* RT-q-PCR.

**Knockdown of *Lcn2* by lentivirus system**

Three short hairpins (sh) RNA (NCBI accession number of the gene sequences used for the LCN2 was NM_008491.1. The shRNA sequences were listed below: shLCN2-a: CGCTACTGGATCAGAACATTT; shLCN2-b: GCCAGTTCACTCTGGGAAATA; shLCN2-c: CCAGGACTCAACTCAGAACTT) were designed and synthesized, which was then ligated into lentiviral backbone LV-U6-CMV/EGFP/F2A/Puro(OBIO Biosciences, Inc). These plasmids were used to produce lentivirus in HEK-293T cells with the packaging plasmids pCMV-VSVG, pMDLg/pRRE, and pRSV-REV. The supernatant containing infectious lentiviruses was harvested at 48 h post-transfection and filtered through 0.45 μm PVDF filters. The virus was concentrated 100-fold by ultracentrifugation (2 h at 60,000 g) and the virus-containing pellet was dissolved in HBSS. When reached 60% confluence, CD24^+^LCN2^+^ LPCs were infected with recombinant lentiviruses (MOI of lv particles=10) for 6-8 hours at 37ºC, together with a lentiviral transfection enhancer (Sigma-Aldrich).

**Quantitative real-time PCR (RT-q-PCR)**

The total RNA of cells was extracted using TRIzol reagent (Invitrogen) according to the manufacturer’s protocols. RT-q-PCR analyses were performed using a LightCycler® 96 Real-Time PCR System (Roche) and SYBR Green PCR kit (Roche). Gene transcription was evaluated using the ΔΔCt method normalized to the housekeeping gene *actin beta (Actin)*. Primer sequences are respectively listed in Table S1.

**Flow cytometry**

For flow cytometry, LPCs were incubated with conjugated antibodies (Additional file 1 Table 3: Antibodies list), at 4 °C for 30 min, or were fixed with Fixation and Permeabilization Solution (BD, 6292704) at 4 °C for 20 min and then incubated with primary antibodies (Additional file 1 Table 3: Antibodies list), followed by staining with secondary antibodies. Then cells were washed twice with the staining buffer and subjected to analysis.

**Cell counting with CCK-8 & EdU assay**

To evaluate cell proliferation, 1,000 cells were seeded into 96-well Matrigel-coated plates for 12 h and then with a medium containing 10% (v/v) CCK-8 (Dojindo) for 1 h. Testing was carried out every 12 h. Proliferation was determined by absorbance measurement at 450 nm using a multimode reader Synergy2 (BioTek). EdU detection was performed using the Cell-Light™ EdU Apollo®488 In Vitro Imaging Kit (RiboBio, Guangzhou). Cells stained at passage 5, were mounted with 1 × Hoechst 33342 and photographed with Leica TCS SP8.

**Additional file 1 Figures and Tables**

**
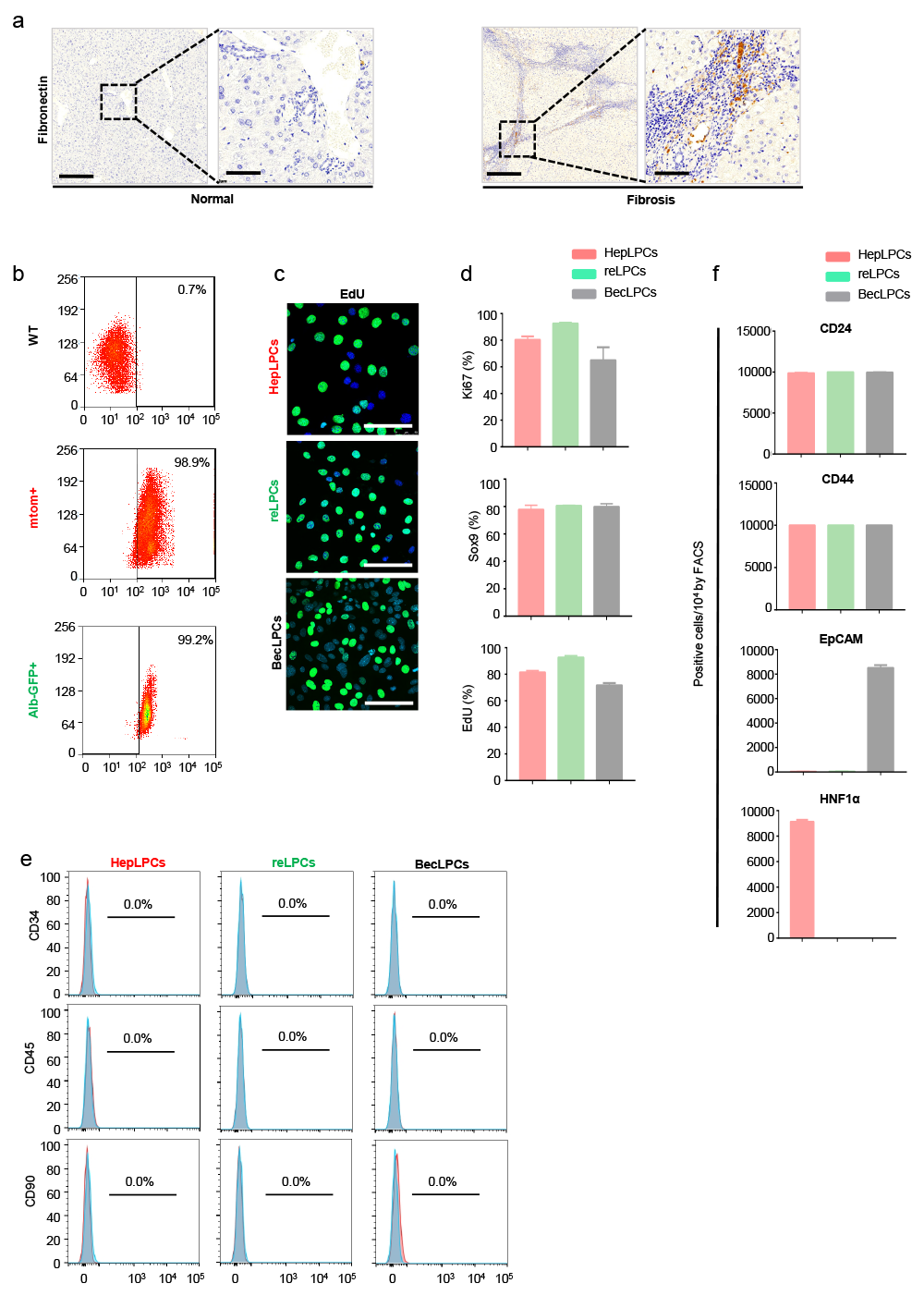
**

**Additional file 1 Figure 1:**

1. Representative immunohistochemical staining of the Fibronectin expression levels in human liver samples. Scale bars, 600 μm (left); 200 μm (right).
2. Flow cytometric analysis showing the proportion of tomato or GFP positive cells in the populations.
3. Representative immunofluorescence staining of EdU (Green) in three cultured LPCs at passage 5 in TEM. Scale bars: 50 μm.
4. Quantification of EdU, Ki67, and Sox9 positive cells in three cultured LPCs at passage 5 in TEM.
5. Quantification of CD34, CD45, and CD90 positive cells among three cultured LPCs at passage 5, assessed by flow cytometry. Red peaks represent staining samples and blue peaks represent the isotype control.
6. Quantification of HNF1α, EpCAM, Cd24 and Cd44 positive cells, measured by FACS analysis.

Resident LPCs-derived LPCs, reLPCs; Hepatocytes-derived LPCs, HepLPCs; BECs-derived LPCs, BecLPCs.

**
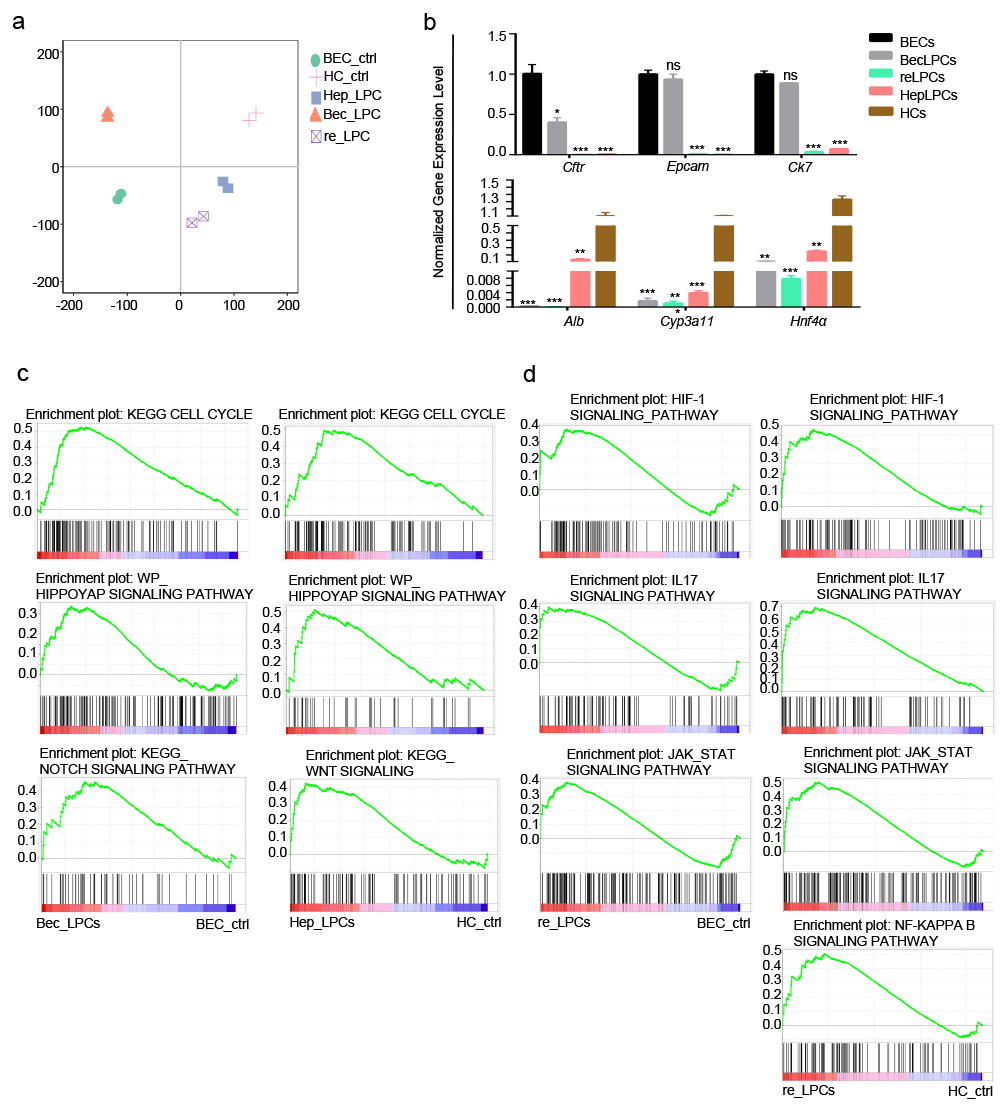
**

**Additional file 1 Figure 2**

1. Principal-component analysis (PCA) was performed to compare global gene expression profiles in three cultured LPCs, hepatocytes (HC), and biliary epithelial cells (BEC). Each element represents the log2 (normalized expression), as scaled by the corresponding color legends. *n* = 2 independent experiments.
2. RT-q-PCR analyses of hepatic and progenitor marker genes in three cultured LPCs, hepatocytes (HC), and biliary epithelial cells (BEC). The data are expressed as the means ± SD of three independent experiments. **P* < 0.05; ***P* < 0.005; ****P* < 0.001; ns represents no significance.
3. GSEA plot showing the relative expression of genes associated with cell cycle, HIPPO, WNT, and NOTCH pathways. BecLPCs were compared with primary BECs, and HepLPCs were compared with primary HCs.
4. GSEA plot showing the relative expression of genes associated with HIF-1, IL17, JAK-STAT, PI3K, and NFkB pathways. Resident LPC-derived LPCs were compared with primary BECs and HCs.

Resident LPCs-derived LPCs, reLPCs; Hepatocytes-derived LPCs, HepLPCs; BECs-derived LPCs, BecLPCs.


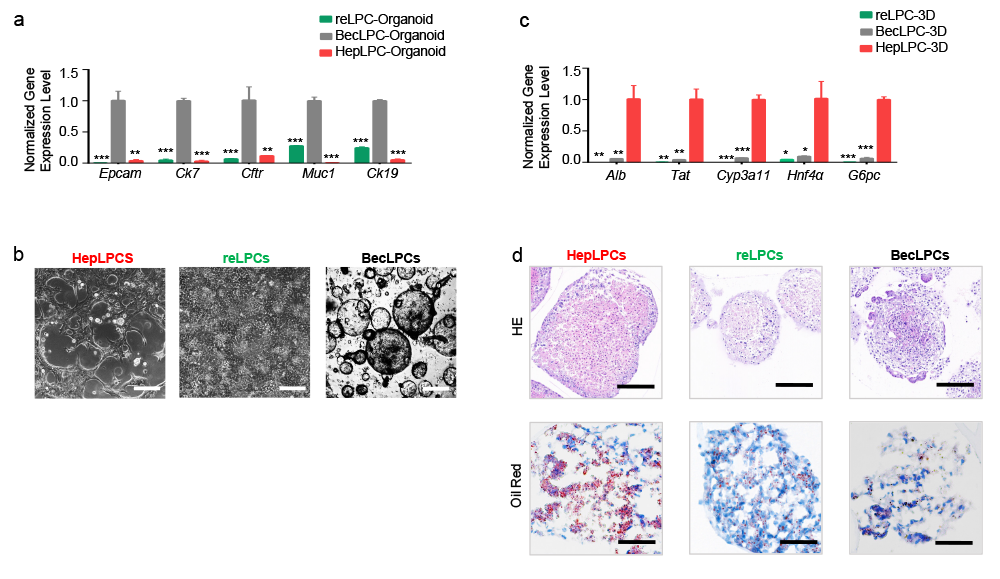


**Additional file 1 Figure 3**

1. Normalized expression levels of bile duct markers genes in LPCs derived organoids, analyzed by RT-q-PCR.
2. Representative images of organoids formed by three cultured LPCs. Scale bars, 200 μm.
3. Normalized expression levels of hepatic and progenitor marker genes in LPCs derived organoids, analyzed by RT-q-PCR.
4. Representative H&E and Oil Red staining in 3D spheroid formed by LPCs at day 10. Scale bar, 100 μm.

Resident LPCs-derived LPCs, reLPCs; Hepatocytes-derived LPCs, HepLPCs; BECs-derived LPCs, BecLPCs. For a and c, The data are expressed as the means ± SD of three independent experiments. **P* < 0.05; ***P* < 0.005; ****P* < 0.001; ns represents no significance.


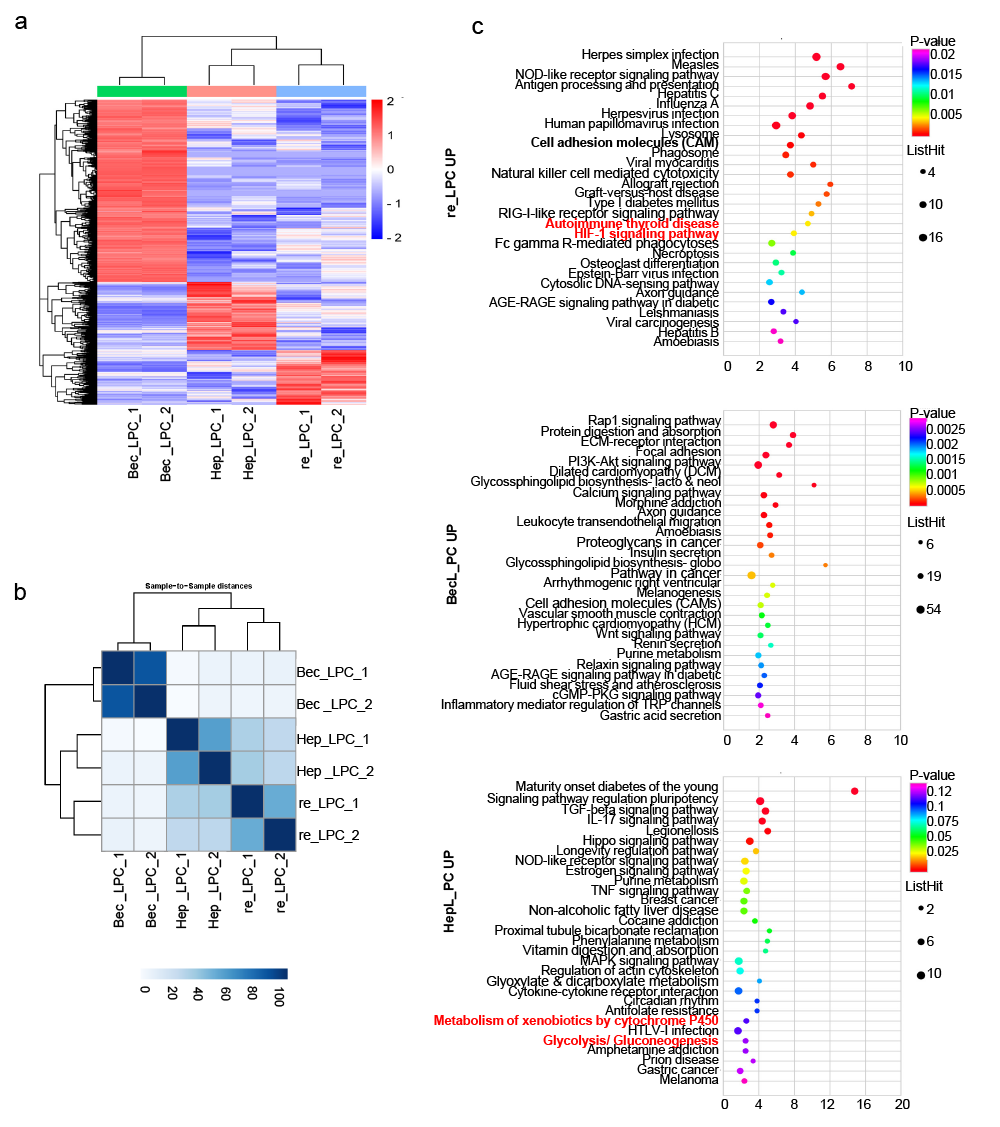


**Additional file 1 Figure 4**

1. Heatmap of the tissue-specific genes upregulated in BecLPCs compared to both HepLPCs and reLPCs (*n*=1362 genes), HepLPCs compared to both BecLPCs and reLPCs (*n*=308 genes), and reLPCs compared to both HepLPCs and BecLPCs (*n*=278 genes).
2. Heatmap of sample-to-sample distance matrix using Poisson distance with hierarchical clustering, depicting overall similarity of transcriptome profiles of three cultured LPCs. The color scale indicates Poisson distance values between samples.
3. Enrichment analysis of the top 30 KEGG pathways in the intersection of the genes in three cultured LPCs, as indicated by the color legend.

Resident LPCs-derived LPCs, reLPCs; Hepatocytes-derived LPCs, HepLPCs; BECs-derived LPCs, BecLPCs.

**
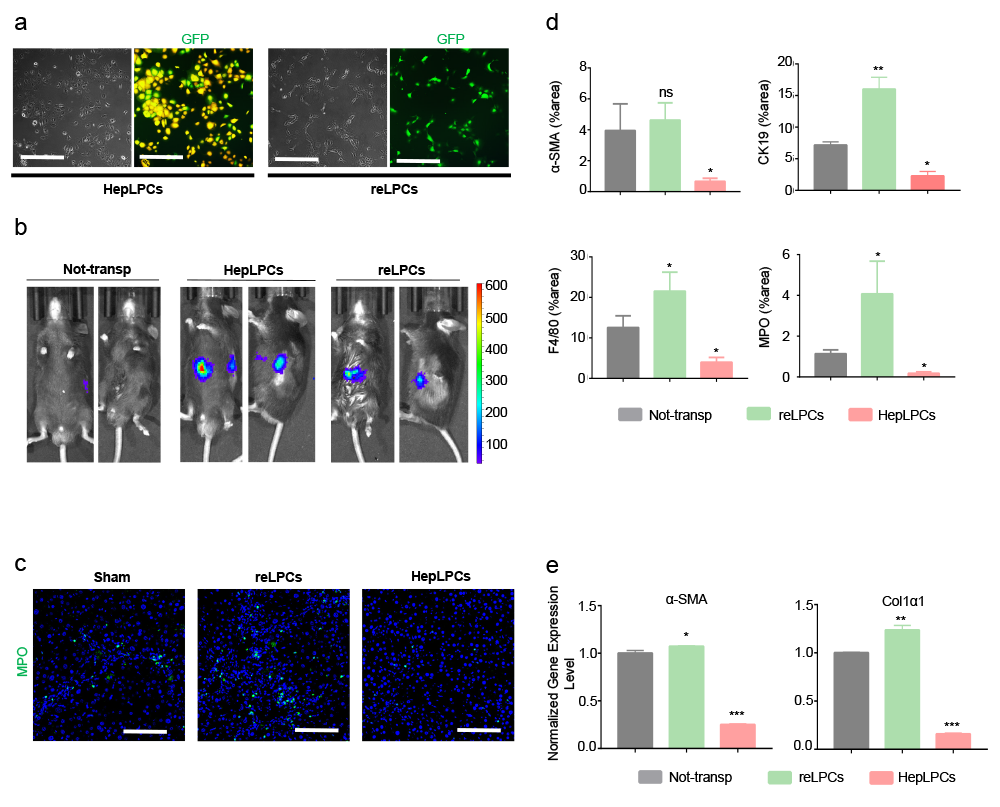
**

**Additional file 1 Figure 5**

1. HepLPCs (tdtom^+^) and CD24^+^LCN2^+^ LPCs were stably transfected with a lentivirus vector carrying luciferase, photomicrograph phase contrast (left), and GFP fluorescence (right). Scale bars, 200μm.
2. Live images of mice 24 hours after transplantation of HepLPCs or CD24^+^LCN2^+^ LPCs.
3. Representative immunofluorescent staining of MPO in the liver slide. Scale bars, 200 μm.
4. Quantification of positive-staining areas for α-SMA, CK19, F4/80, and MPO were measured by Image J software.
5. RT-q-PCR analyses of the expression levels of α-SMA and Col1α1 in the fibrotic liver of mice.

Resident LPCs-derived CD24^+^LCN2^+^ LPCs, reLPCs; Hepatocytes-derived LPCs, HepLPCs. For d and e, the data are expressed as the means ± SD of three independent experiments. **P* < 0.05; ***P* < 0.005.


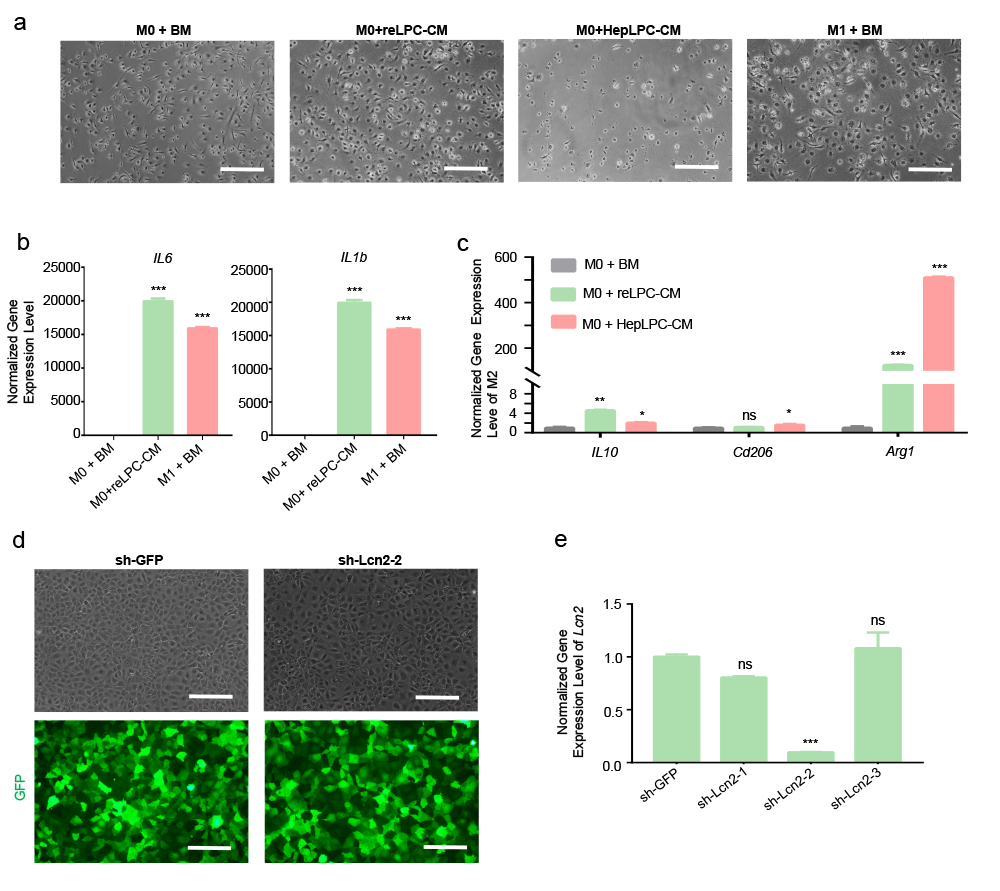


**Additional file 1 Figure 6**

1. The BMDMs were stimulated with LPS/IFN-γ or conditioned mediums (CM) of HepLPCs or CD24^+^LCN2^+^ LPCs for M1 polarization, respectively, for 24 h. The morphology of the BMDMs was observed under a microscope. Scale bars, 200μm.
2. RT-q-PCR analyses of the expression levels of M1 macrophages, M1 and M0 macrophages were cultured in the basal medium (BM) or CM of CD24^+^LCN2^+^ LPCs.
3. RT-q-PCR analyses of the expression levels of M2 macrophages. M0 macrophages were cultured in the basal medium (BM) or indicated conditioned medium (CM) of CD24^+^LCN2^+^ LPCs or HepLPCs.
4. CD24^+^LCN2^+^ LPCs were stably transfected with a lentivirus carrying sh-LCN2 or sh-GFP. Photomicrographs are shown as phase-contrast (up), and GFP fluorescence (down). Scale bars, 200 μm.
5. RT-q-PCR analyses of the expression levels of LCN2 expression in CD24^+^LCN2^+^ LPCs with sh-LCN2 or sh-GFP.

Resident LPCs-derived CD24^+^LCN2^+^ LPCs, reLPCs; Hepatocytes-derived LPCs, HepLPCs. For panels b, c, and e, the data are expressed as the means ± SD of three independent experiments. **P* < 0.05; ***P* < 0.005; ****P* < 0.001; ns represents no significance.

**
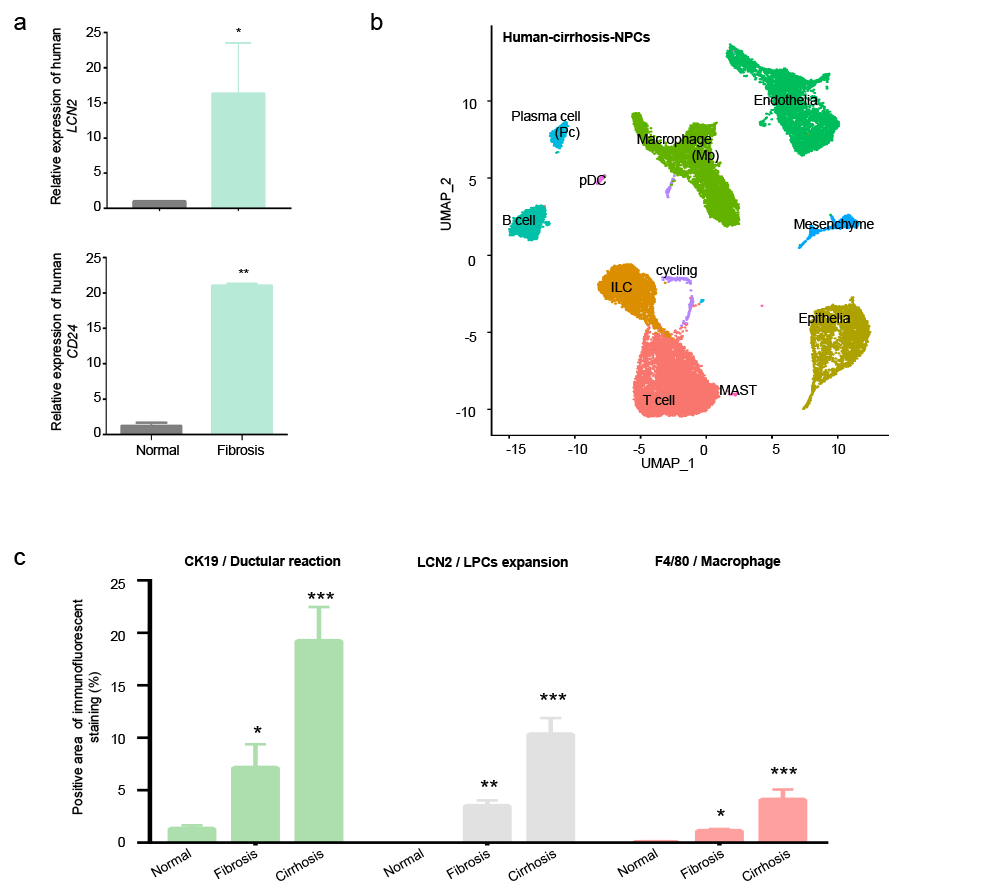
**

**Additional file 1 Figure 7**

1. RT-q-PCR analysis of *LCN2* and *CD24* expression in human liver diagnosed with fibrosis. The data are expressed as the means ± SD of three independent experiments. **P* < 0.05; ***P* < 0.005; ****P* < 0.001.
2. Liver non-parenchymal cells (NPCs) were isolated from human liver cirrhosis tissues and subjected to scRNA-Seq. U-map visualization of liver cell clusters based on 25477 single-cell transcriptomes.
3. Quantification of positive-staining areas for CK19, F4/80, and LCN2 were measured by Image J software.

For a and c, the data are expressed as the means ± SD of three independent experiments. **P* < 0.05; ***P* < 0.005; ****P* < 0.001; ns represents no significance.

**Additional file 1 Table 1: Primer list.**

| **Gene** | **Forward sequence 5’->3’** | **Reverse sequence 5’->3’** |
| --- | --- | --- |
| *Cd24* | GTTGCACCGTTTCCCGGTAA | CCCCTCTGGTGGTAGCGTTA |
| *Ck19* | GTTCAGTACGCATTGGGTCAG | GAGGACGAGGTCACGAAGC |
| *Ck7* | AGGAGATCAACCGACGCAC | GTCTCGTGAAGGGTCTTGAGG |
| *Epcam* | CTGGCGTCTAAATGCTTGGC | CCTTGTCGGTTCTTCGGACTC |
| *Alb* | CAAGAGTGAGATCGCCCATCG | TTACTTCCTGCACTAATTTGGCA |
| *Cyp3a11* | CCTGGGTGCTCCTAGCAATC | CAAGGAGAGGCGTTTGACCA |
| *Cftr* | CCCTTCGGCGATGCTTTTTC | AAGCCTATGCCAAGGTAAATGG |
| *Tat* | TGCTGGATGTTCGCGTCAATA | CGGCTTCACCTTCATGTTGTC |
| *G6PC* | CGACTCGCTATCTCCAAGTGA | GGGCGTTGTCCAAACAGAAT |
| *Actin* | ATGCCACAGGATTCCATACCCAAG | CTCTAGACTTCGAGCAGGAGATGG |
| *HNF4α* | ATGCGACTCTCTAAAACCCTTG | ACCTTCAGATGGGGACGTGT |
| *α-SMA* | CCCAGACATCAGGGAGTAATGG | TCTATCGGATACTTCAGCGTCA |
| *COLLα1* | ATCGGTCATGCTCTCTCCAAACCA | ACTGCAACATGGAGACAGGTCAGA |
| *Sox9* | AGTACCCGCATCTGCACAAC | ACGAAGGGTCTCTTCTCGCT |
| *Krt17* | ACCATCCGCCAGTTTACCTC | ACTACCCAGGCCACTAGCTG |
| *Nrp2* | GCTGGCTACATCACTTCCCC | GGGCGTAGACAATCCACTCA |
| *Parp14* | AGCAGTGGATCAGAAAAGACAG | GTCAGCACCATCTCGGATACT |
| *Oas2* | CGCTCCAATGAGAATCCCAG | TTCCAGTGCGTACAGCAATGG |
| *Onecut2* | ACACCACGCCATGAGTATGTC | CGAAATTGGGGCTGAGCATTTT |
| *Lgals2* | AACATGAAACCAGGGATGTCC | CGAGGGTTAAAATGCAGGTTGAG |
| *Rbp4* | ACGAGTCCGTCTTCTGAGCA | TGGTCATCGTTTCCTCGCTG |
| *Stra6* | GAGTCCCAGGCATCTGAGAAT | CCAGGAACGACAGTGAAGCC |
| *Ltbp2* | GCTCACCGGGAGAAATGTCTG | CAGGTTTGATACAGTGGTTGGT |
| *Lcn2* | GGGAAATATGCACAGGTATCCTC | CATGGCGAACTGGTTGTAGTC |
| *Lgr6* | ATCATGCTGTCCGCTGACTG | ACTGAGGTCTAGGTAAGCCGT |
| *Nlrc5* | GCTGAGAGCATCCGACTGAAC | AGGTACATCAAGCTCGAAGCA |
| *Hnf1a* | GACCTGACCGAGTTGCCTAAT | GCGAAGTCTTCCCCATCGTC |
| *H-Lcn2* | CCACCTCAGACCTGATCCCA | CCCCTGGAATTGGTTGTCCTG |
| *H-Cd24* | CTCCTACCCACGCAGATTTATTC | AGAGTGAGACCACGAAGAGAC |
| *ACTB* | GCCTCGCTGTCCACCTTCC | TGCTGTCACCTTCACCGTTCC |

**Additional file 1 Table 2: Abbreviations.**

| CD24 | cluster of differentiation 24 |
| --- | --- |
| HNF4α | hepatocyte nuclear factor 4-alpha |
| CK19 | Cytokeratin 19 |
| ALB | albumin |
| CYP3a11 | cytochrome P450 family 3 subfamily A member 11 |
| CYP1a2 | cytochrome P450 family 1 subfamily A member 2 |
| Tat | tyrosine aminotransferase |
| G6PC | glucose-6-phosphatase, catalytic |
| Sox9 | SRY (sex determining region Y)-box 9 |
| EpCAM | epithelial cell adhesion molecule |
| α-SMA | alpha smooth muscle Actin |
| COL1α1 | collagen type I alpha 1 chain |
| HepLPCs | hepatocyte-derived liver progenitor cells |
| reLPCs | Resident LPCs-derived liver progenitor cells |
| BecLPCs | BECs-derived liver progenitor cells |
| LPCs | liver progenitor-like cell |
| NPCs | nonparenchymal cells |
| tdtom | tdtomato |
| GFP | Green fluorescent protein |
| H&E | hematoxylin eosin |
| ELISA | enzyme-linked immunosorbent assay |
| CCl_4_ | carbon tetrachloride |
| TEM | Transition and Expansion Medium |
| TBG | thyroxine-binding globulin |
| HCs | Hepatocytes |
| BECs | Biliary epithelial cells |
| FACS | fluorescence-activated cell sorting |
| DAPI | 4’,6-diamidino-2-phenylindole |
| Fah | fumarylacetoacetate hydrolase |
| FoxA | helix/forkhead box |
| MACS | magnetic activated cell sorting |
| LCN2 | lipocalin-2 |

**Additional file 1 Table 3: Antibodies list**

| **Antibody** | **Company** | **Product code** | **Conjugate** | **Dilution** |
| --- | --- | --- | --- | --- |
| ALB | proteintech | 16475-1-AP | **-** | 1:200 |
| CYP3A4 | proteintech | 18227-1-AP | **-** | 1:200 |
| LCN2 (human) | proteintech | 26991-1-AP | **-** | 1:200 |
| LCN2 (mouse) | R&D | AF1857 |  | 1:200 |
| fibronectin | proteintech | 15613-1-AP | **-** | 1:200 |
| CK19 | proteintech | 10712-1-AP | **-** | 1:200 |
| EpCAM | Abcam | ab71916 | **-** | 1:200 |
| FAH | Abcam | ab151998 | **-** | 1:100 |
| SOX9 | Sigma-Aldrich | AB5535 |  | 1:100 |
| HNF1A | Santa cruz | sc-393925 |  | 1:100 |
| αSMA | Abcam | ab124964 | **-** | 1:200 |
| CD24 (mouse) | Biolegend | 101801 | **-** | 1:200 |
| CD24 (human) | NOVUS | NB100-64861 | **-** | 1:100 |
| Ki67 | Abcam | ab16667 | **-** | 1:200 |
| EpCAM | Biolegend | 118213 | APC | 1:100 |
| CD24 | Biolegend | 101814 | APC | 1:100 |
| CD44 | BD Pharmingen | 561862 | APC | 1:100 |
| CD34 | BD Pharmingen | 560238 | FITC | 1:100 |
| CD45 | BD Pharmingen | 567377 | 488 | 1:100 |
| CD90 | BD Pharmingen | 554894 | FITC | 1:100 |
| HNF4A | Abcam | Ab41898 | - | 1:200 |

**Additional file 1 Table 4: Age and gender of the 9 patients with clinically diagnosed liver fibrosis and cirrhosis after Hepatitis B infection.**

| **Patients Number** | **Age** | **Gender** |
| --- | --- | --- |
| 1 | 78 | male |
| 2 | 51 | male |
| 3 | 75 | male |
| 4 | 56 | male |
| 5 | 50 | male |
| 6 | 57 | male |
| 7 | 67 | male |
| 8 | 60 | male |
| 9 | 41 | male |

**Additional file 1 Table 5: Age and gender of the 5 patients with clinically diagnosed hepatic hemangioma.**

| **Patients Number** | **Age** | **Gender** |
| --- | --- | --- |
| 1 | 49 | male |
| 2 | 50 | male |
| 3 | 63 | male |
| 4 | 61 | male |
| 5 | 32 | female |

**References**

1. Huang WJ, Zhou X, Fu GB, Ding M, Wu HP, Zeng M, et al. The combined induction of liver progenitor cells and the suppression of stellate cells by small molecules reverts chronic hepatic dysfunction. Theranostics. 2021;11(11):5539-52.

2. Fu GB, Huang WJ, Zeng M, Zhou X, Wu HP, Liu CC, et al. Expansion and differentiation of human hepatocyte-derived liver progenitor-like cells and their use for the study of hepatotropic pathogens. Cell Res. 2019;29(1):8-22.

3. Hu H, Gehart H, Artegiani B, C LO-I, Dekkers F, Basak O, et al. Long-Term Expansion of Functional Mouse and Human Hepatocytes as 3D Organoids. Cell. 2018;175(6):1591-606 e19.

4. Ma PF, Gao CC, Yi J, Zhao JL, Liang SQ, Zhao Y, et al. Cytotherapy with M1-polarized macrophages ameliorates liver fibrosis by modulating immune microenvironment in mice. J Hepatol. 2017;67(4):770-
